# Acoustic telemetry for marine animal and environmental monitoring: Technologies, challenges, and emerging trends

**DOI:** 10.1016/j.fmre.2026.05.019

**Published:** 2026-07-24

**Authors:** Abdelrahman Samy Ismail, Zhenting Ye, Amar N. Alsheavi, Yaxiaer Yalikun, Yang Yang, Chenjing Shang

**Affiliations:** a Shenzhen Key Laboratory of Marine Bioresource and Eco-Environmental Science, College of Life Sciences and Oceanography, Shenzhen University, Shenzhen 518060, China; b Institute of Deep-Sea Science and Engineering, Chinese Academy of Sciences, Sanya 572000, China; c Faculty of Science, Zagazig University, Zagazig 44519, Egypt; d University of Chinese Academy of Sciences, Beijing 100049, China; e School of Computer Science and Technology, University of Science and Technology of China, Hefei 23002, China; f Division of Materials Science, Nara Institute of Science and Technology, 8916-5 Takayama-cho, Ikoma, Nara 630-0192, Japan

**Keywords:** Acoustic telemetry, Marine animal tracking, Ocean monitoring, Acoustic tags, Ocean energy harvesting, Flexible electronics, Marine conservation

## Abstract

Acoustic telemetry is a fundamental tool for tracking marine animals, providing long-term data that support fisheries management, conservation, and ecosystem-based monitoring. Advances in acoustic tags, receiver arrays, and sensor-integrated transmitters have expanded the ability to quantify animal movement, habitat use, and physiological responses across diverse marine environments. However, the long-term deployment of telemetry systems remains constrained by limited power supply, device size, detection performance in complex habitats, and environmental interference. This review provides an in-depth analysis of the fundamental principles and system architectures of acoustic telemetry, with emphasis on tag design, sensing capabilities, positioning approaches, and data workflows. It further highlights major marine monitoring applications, including pollution impact assessment, climate change response, marine protected area (MPA) design, and combating illegal, unreported, and unregulated (IUU) fishing. To address persistent energy and durability limitations, the review synthesizes emerging advances in sustainable power strategies, including flexible, self-healing, stretchable, and biodegradable energy-harvesting and storage materials, as well as ocean-wave energy-harvesting technologies for autonomous, long-term deployment. By linking recent progress in acoustic telemetry with sustainable energy solutions, this paper identifies current challenges and future directions for resilient, environmentally responsible, next-generation marine observation platforms.

## 1 Introduction

The ocean is vital for Earth’s ecological and climate stability, supporting biodiversity, regulating temperatures, and providing resources for human societies. It supplies 16% of global animal protein, with coastal communities relying on fisheries for 20% of their dietary protein, feeding 3.3 billion people [1,2]. Sustainable marine practices could meet the nutritional needs of 10 billion by 2050 [3]. However, anthropogenic pressures, overfishing, habitat loss, and climate change are degrading these ecosystems at alarming rates [4]. This erosion of marine biodiversity directly threatens essential services, like carbon sequestration and coastal protection [5]. Restoring oceanic resilience is now both an ecological imperative and a socioeconomic necessity.

Acoustic telemetry is key for studying aquatic ecosystems, as it tracks aquatic animals by attaching or implanting small transmitters and using receiver networks to monitor their behavior, physiology, and movements [6]. It applies to small or bottom-dwelling species, such as turtles and lobsters [7,8], and to large migratory species, such as sharks and tuna that traverse entire ocean basins [9,10]. Acoustic telemetry operates through: (1) sound-emitting tags, (2) stationary or mobile underwater receivers that detect signals, and (3) data processing algorithms that decode movement paths and behavioral patterns. This system enables tracking of individual organisms over long periods across various marine environments, from freshwater to the open ocean, using active (real-time) or passive (fixed) tracking. Acoustic telemetry can be categorized by tag functionality into three types: transmitter-only tags (broadcast-only), transceiver tags (two-way communication), and sensor-integrated tags that log environmental data. These configurations support applications from basic movement tracking to monitoring physiological parameters like breathing rates in farmed fish [11] and environmental interactions in seaweed habitats [12]. It provides high-resolution, long-term data for modeling species distribution, behavior, and environmental impacts, including effects of renewable energy infrastructure [[13], [14], [15]]. Its versatility is demonstrated through monitoring fish in dams, fisheries, and cages [16,17], tracking the marine environment with underwater vehicles [18,19], and aquaculture cage detection [20]. A comprehensive acoustic telemetry network (see Fig. 1) supports ocean monitoring through four stages. First, environmental challenges shape sensing needs and drive the development of complex underwater systems. Second, advancements in acoustic telemetry, from moored to cabled networks, enable high-resolution data collection. Third, these data inform species management by revealing movement, behavior, and habitat use. Fourth, ecological insights promote better bio-tagging, more durable, miniaturized, with advanced sensors and communication. This cycle enhances ocean monitoring, increasing transparency and sustainability. The core of Fig. 1 illustrates the methods forming a multi-scenario marine monitoring system. Coastal areas use passive arrays to detect signals from tagged dolphins and fish for tracking and habitat studies. Offshore platforms and vessels gather position data via satellite. Aquaculture monitors fish health with environmental sensors and satellite modules. Trawling vessels tow nets with automated tags for regional population, acoustic, and environmental data. Deep-sea monitoring employs AUVs and ROVs with hydrophones to detect tagged species, like crustaceans, where fixed receivers are unavailable. Surface animals, like whales and sea turtles, carry bio-logging tags to record sound, posture, depth, and environment, and transmit data when they surface or breathe. These tools measure various parameters, supporting ecological research, species management, and ocean studies.

**Fig. 1:**
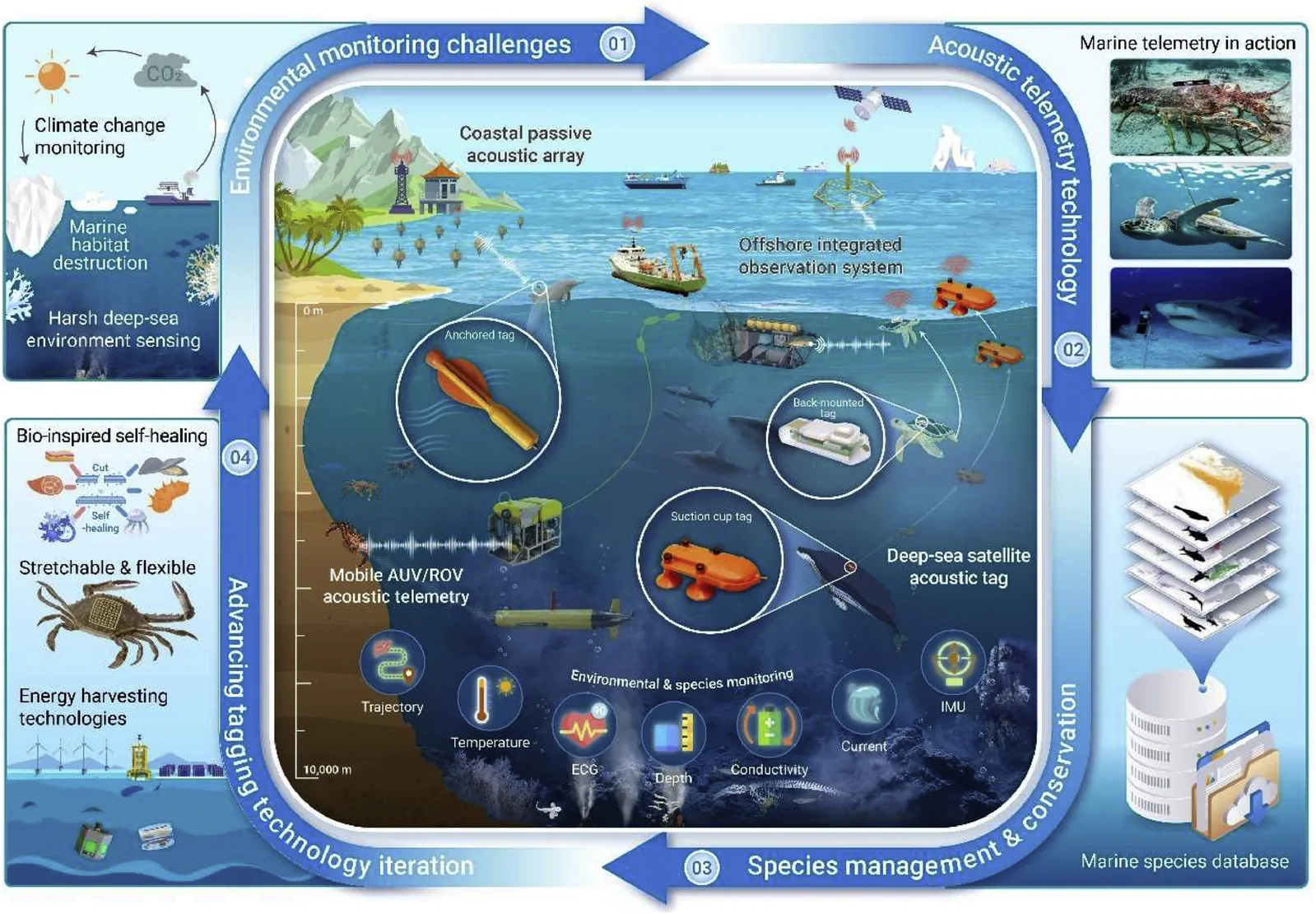
**A full-scenario acoustic telemetry and sensing network seeks transparent oceans.** It works in an iterative cycle: (1) environmental monitoring challenges identify sensing needs for ocean observation, leading to (2) development of acoustic telemetry systems to track animals and collect environmental data across coastal, pelagic, and deep-sea areas [9]. Copyright 2015, American Association for the Advancement of Science. (3) Telemetry data support species management and conservation, revealing movement, habitat use, and ecological interactions [29]. Copyright 2011, Springer Nature Limited. These insights guide (4) improvements in bio-tagging technologies, including sensors, attachment methods, power systems, and data transmission [[30], [31], [32], [33]]. The enhanced tags then feed back into the monitoring network, strengthening its capacity and closing the loop toward a more transparent ocean.

However, despite its potential, deploying acoustic telemetry systems remains challenging due to power limitations, signal attenuation in complex environments, and the ecological impacts of electronic tagging. Traditional approaches to address power constraints have focused on low-power designs [11,12], tag miniaturization [[21], [22], [23]], or power management [24,25]. While these approaches extend battery life, they remain constrained by the challenges of replacing or recharging batteries in remote marine environments [[26], [27], [28]]. To address these limitations, recent advances in ocean energy harvesting from waves, currents, and other marine sources are transforming underwater monitoring [[34], [35], [36]]. Integrating energy harvesting methods like triboelectric nanogenerators (TENGs) [32,[37], [38], [39], [40]], piezoelectric generators [26,[41], [42], [43]], electromagnetic generators [44], and biodegradable batteries [[45], [46], [47], [48]] into acoustic telemetry and sensor networks can extend tag longevity, reduce maintenance, and minimize environmental impact [[49], [50], [51], [52], [53]]. These innovations enable self-powered, maintenance-free, and eco-friendly tracking systems for remote marine environments. For instance, energy-harvesting transmitters utilizing fish movement or ambient ocean energy have enabled the first battery-free, long-term animal tracking studies, opening new avenues for behavioral research and environmental impact assessment [27,54]. Furthermore, emerging trends in miniaturization, machine learning-enhanced data processing, and multi-sensor fusion are expanding acoustic telemetry beyond simple movement tracking to include physiological and environmental monitoring.

Although several reviews have explored acoustic telemetry applications in various contexts [9,25,[55], [56], [57], [58]], ocean energy harvesting independently [39,42,49,59,60], or flexible and biodegradable energy harvesting and storage [46,48,[61], [62], [63], [64], [65]], there remains a significant gap persists in the systematic approach to acoustic telemetry architectures from first principles, particularly concerning sustainability and long-term independence. Most existing surveys focus either on technical implementation details or ecological applications, without thoroughly exploring how these systems can be co-designed with emerging energy and materials technologies to ensure sustainable and environmentally responsible ocean observation networks [26,49,53]. To address this gap, this review provides an in-depth analysis of the fundamental principles and system architectures of acoustic telemetry, along with a structured overview of its applications for monitoring marine and freshwater animals. It begins with the fundamental working principles, including transmitter–receiver setups, communication methods, and signal processing. It then examines acoustic telemetry architectures and tag design by classifying tags according to operational capability (transmitters, transceivers, and sensor-integrated tags) and comparing their trade-offs in power consumption, communication flexibility, sensing capacity, deployment duration, and ecological impact. Building on this technical foundation, the review highlights four major marine monitoring applications: pollution impact assessment, climate change response, marine protected area (MPA) design, and combating illegal, unreported, and unregulated (IUU) fishing. Finally, it discusses key limitations and shows how emerging advances, particularly flexible energy-harvesting/storage systems and ocean-wave energy harvesting, can help overcome battery constraints and support long-term or near-continuous telemetry deployment. The main contributions of this review are as follows:

• Providing an in-depth analysis of the fundamental principles and system architectures of acoustic telemetry, including tag-receiver configurations, communication protocols, network deployment strategies, and the key physical constraints that affect detection performance in marine and freshwater environments.
• Providing a pragmatic framework for the architecture and design of acoustic telemetry systems by classifying acoustic tags into transmitters, transceivers, and sensor-integrated variants, while comparing their trade-offs concerning power consumption, sensing capability, communication flexibility, deployment duration, and ecological impact.
• Exploring the primary applications of acoustic telemetry in pollution assessment, climate change impact studies, MPA design, and IUU fishing detection, and subsequently linking these requirements to emerging advancements in sustainable energy and materials. These advancements include flexible, self-healing, stretchable, biodegradable energy systems, as well as ocean-wave energy harvesting for long-term, autonomous deployment.

By explicitly linking acoustic telemetry technologies with emerging energy and materials solutions, this review provides a comprehensive reference to advance next-generation, sustainable, and scalable aquatic monitoring systems for research, conservation, and fisheries governance.

## 2 Fundamentals of acoustic telemetry

Acoustic telemetry is a widely used technique for studying the movement and spatial ecology of aquatic animals across diverse environments, ranging from freshwater systems to open oceans and from polar to tropical regions [9,55]. It uses stationary or mobile acoustic receivers to detect signals from tags attached to animals, enabling the collection of presence and location data over extended spatial and temporal scales. Despite variations in system configurations, tag designs, and deployment protocols, the fundamental objective of acoustic telemetry is to provide reliable, high-resolution information on animal movement and habitat use. Over the past decades, this technology has led to significant discoveries, improving our understanding of aquatic ecosystems [7]. It has revealed migratory patterns of anadromous fish [[66], [67], [68]], predator-prey interactions [69,70], post-release survival of bycatch [8,71], and key demographic data for fisheries management [24,72,73]. Its operation across spatial scales (meters to a few kilometers) and temporal scales (days to years) helps in understanding ecological responses to human impacts like invasive species, migration barriers, habitat loss, and climate change [74]. Moreover, it supports conservation efforts and fisheries management by identifying key habitats, migration routes, and guiding policies such as fishing quotas, transboundary species management, and protected areas [25].

The following subsections introduce the fundamental working principles of acoustic telemetry and place them in context by comparing this approach with alternative animal tracking technologies, thereby establishing the foundation for subsequent discussions on system architectures, applications, and emerging technological advances.

### 2.1 Acoustic telemetry working principles

Acoustic telemetry systems function via a signal chain comprising acoustic signal generation at the tag, underwater propagation, signal detection by receivers, and subsequent post-processing for animal localization and behavioral analysis. Fig. 2a illustrates the fundamental working principle of acoustic telemetry systems. The main components are [66,75]:

**Fig. 2:**
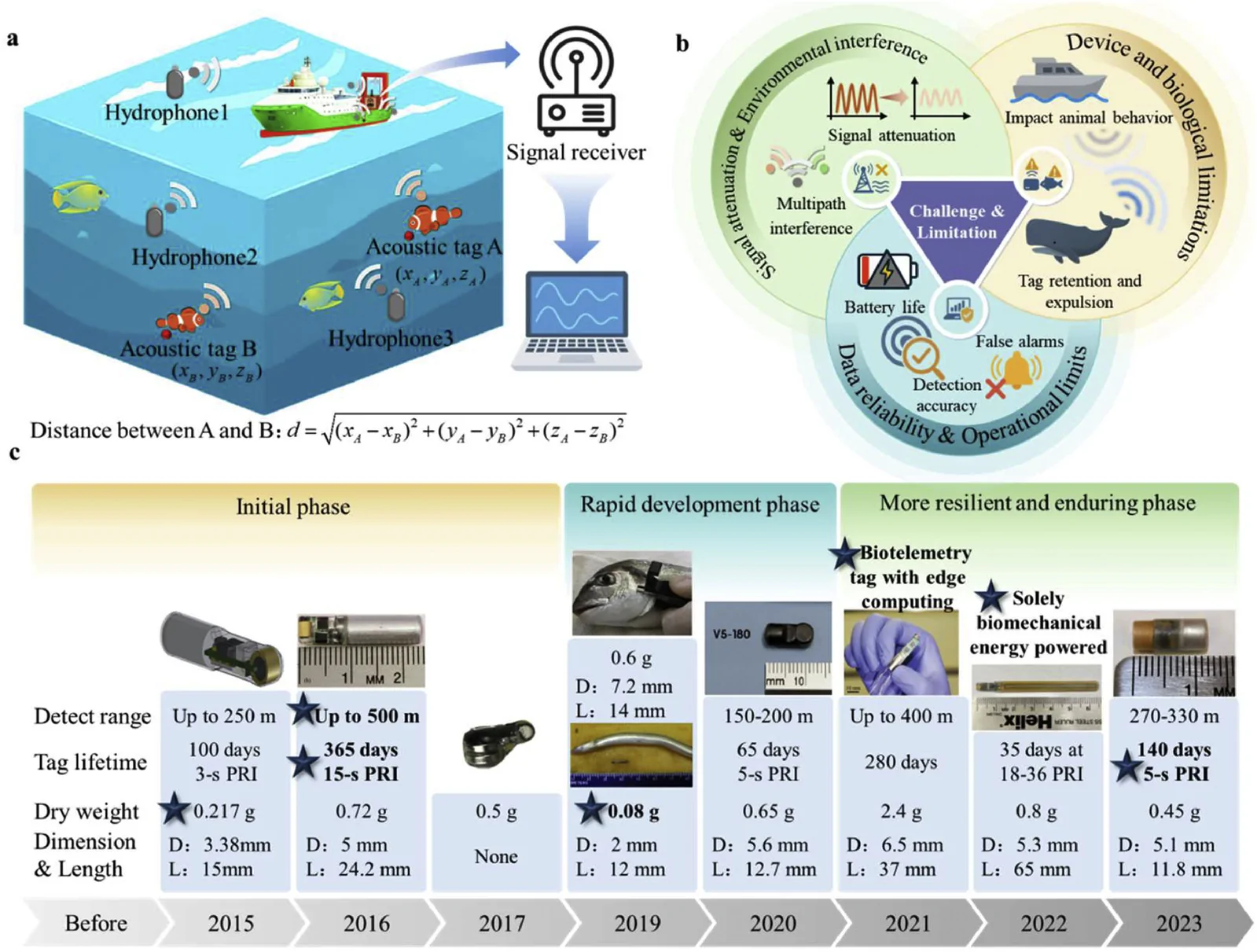
**Overview of acoustic telemetry.** (a) Acoustic telemetry system monitoring of fish trajectory. Points A (x_A_, y_A_, z_A_) and B (x_B_, y_B_, z_B_) represent the fish positions at A and B, respectively, and d represents the distance between them. (b) Challenges and limitations of acoustic telemetry in marine environments include signal attenuation, multipath interference, limited battery life, and background noise. A tagged fish is a tracked marine animal that emits signals detected by surrounding receivers. Environmental and technological constraints are depicted to show the real-world barriers to effective underwater tracking. (c) Timeline of acoustic transmitter development with inset images of key models from 2015 to 2023. Inset images: “First injectable acoustic fish transmitter (JSATS).” [85]. Copyright 2015, Springer Nature. “Sturgeon Transmitter” [86]. Copyright 2016, AIP. “Lotek Mini MAP” [87]. Copyright 2017. “AE-FishBIT” [11]. Copyright 2019, Frontiers. “Tracking juvenile eels and lampreys” [88]. Copyright 2019, MyJove Corporation. “VEMCO V5–1H” [89]. Copyright 2020. “Lab-on-a-fish” [90]. Copyright 2021, IEEE. “Self-powered Transmitter” [91]. Copyright 2022, Elsevier. “Low-frequency Transmitter for Marine Animals” [22]. Copyright 2023, IEEE.

Transmitters (tags) are small devices implanted in or externally attached to aquatic organisms that emit acoustic signals at specific frequencies, typically ranging from 30 kHz to several hundred kHz (up to 416.7 kHz) [57]. Frequency selection depends on the specific application and environmental conditions. Lower frequencies (e.g., 69 kHz) offer longer detection ranges but require larger, more power-consuming transducers, resulting in bulkier tags [57]. In contrast, higher frequencies (307–416.7 kHz) enable smaller tags for small fish (minimizing drag [66]), with better shallow-water resolution, but shorter detection ranges and greater sensitivity to water noise [76]. Acoustic telemetry systems employ different coding schemes to transmit information efficiently. Pulse position modulation (PPM) encodes data by using time intervals between fixed-frequency pulses, whereas code division multiple access (CDMA) incorporates transmitter identification codes directly into the acoustic signal [77]. In CDMA, two modulation schemes are used: binary frequency-shift keying (BFSK), which encodes binary data (0 and 1) via small frequency shifts, and binary phase-shift keying (BPSK), which uses phase shifts for improved noise resistance [66]. For example, BFSK is used in Lotek systems operating at 76 and 200 kHz [78,79], while BPSK is employed in the JSATS system (416.7 kHz) [66] and Innovasea’s HR2 platform (180 kHz) [80].

Different modulation schemes in acoustic telemetry are selected based on environmental conditions and system constraints, particularly regarding noise levels, transmission range, and energy availability. PPM is widely used due to its low power consumption and simple implementation, making it well-suited for long-term deployments in energy-constrained tags [81]. Frequency-based schemes such as BFSK provide improved robustness to acoustic noise and multipath interference, which is critical in complex or dynamic underwater environments [57,82]. Phase-based schemes such as BPSK improve signal reliability and spectral efficiency but require more complex signal processing and higher energy consumption. Consequently, simpler schemes are generally preferred for small, battery-powered transmitters, whereas advanced techniques are used where higher reliability, greater tag density, or bidirectional communication is needed [80,83]. Table 1 summarizes the key characteristics of PPM, BFSK, and BPSK. In shallow waters with lower noise levels, PPM may suffice due to its simplicity and low power consumption, suitable for long-term deployments with fewer tags. In deep, noisy waters where more tags are present, BFSK or BPSK are better due to their robustness and multi-signal handling. Understanding these trade-offs helps optimize telemetry systems in various aquatic monitoring scenarios.

**Table 1:** **Comparison of modulation schemes used in acoustic telemetry systems, highlighting their working principles, advantages, disadvantages, trade-offs in noise resistance, and power consumption**.

Modulation scheme	Working principle	Advantages	Disadvantages	Noise resistance	Power consumption	Best use case	Ref.
PPM (Pulse Position Modulation)	- Encodes data by varying the timing of pulses - Each pulse represents a unique identification code	- Simple implementation - Low power consumption - Require less complex hardware	- Limited number of unique codes - Sensitive to noise	- Low - Less robust in noisy environments	Low	- Shallow water applications - Low tag individuals, - Long-term deployments	[81]
BFSK (Binary Frequency Shift Keying)	- Transmits data by shifting between two frequencies - Representing binary data	- Robust against noise - Maintains signal integrity over long distances	- Lower data transmission rates compared to others - Limit use in applications requiring rapid data updates	Moderate to high	Moderate	- Deep-sea applications - Noisy environments	[57,82]
BPSK (Binary Phase Shift Keying)	- Modulates the phase of a carrier wave to represent binary data - Providing a simple yet effective means of communication	- High noise resistance - Efficient bandwidth utilization - Reliable communication over long distances	- More complex demodulation process - Requiring advanced signal processing methods	High	High	- Suitable for both shallow and deep-sea applications due to its robustness - Long-distance - High noise conditions	[80,83]

Receivers (hydrophones) are devices deployed in water to detect, decode, and store acoustic signals emitted by transmitters within a detection range vary approximately from 100 m to 1 km, depending on water conductivity, temperature, and ambient noise conditions. They can be stationary moorings or mobile platforms that record signal time and strength. Modern acoustic telemetry systems enable high-resolution, long-term monitoring of free-ranging aquatic animals for up to 10 years. Affordable, low-maintenance networks now form the core of fisheries management, with operational lifespans of approximately 5 years, and some models supporting remote data collection via autonomous gliders.

Data processing, following detections, data stored on receivers are sent to a central database for analysis, tracking the movements, behaviors, and habitats of tagged organisms. These data include time-stamped transmitter IDs stored on acoustic receivers. At the end of the studies, data are retrieved by collecting receivers. However, recent technological advancements enable real-time data transmission to investigators. Detection frequency depends on study design and animal behavior; for instance, fast-migrating fish near hydropower stations may be detected every second, whereas lake fish could be detected every few minutes. To monitor fish across vast areas, receivers are organized into extensive arrays covering a few or tens of kilometers [6]. Detection accuracy depends on transmitter range, typically within a few hundred meters. When multiple receivers detect the same transmission, spatial positioning can be achieved using triangulation or time-difference-of-arrival methods, allowing fine-scale movement analysis with sub-meter accuracy. Additionally, modern acoustic tags can include sensors that monitor environmental and physiological data, such as depth, temperature, acceleration, and predation indicators, including pH sensors that detect predator gastric acid [6].

To avoid bias in the results, it is crucial that the fish’s behavior remains unaffected by the transmitter’s additional weight and the tagging method. Although there is no scientific consensus on this issue, a general guideline recommended by the American Fisheries Society suggests that fish should not be equipped with tags that have a dry weight exceeding 2% of the fish’s weight [84]. Finally, Fig. 2c presents a brief timeline illustrating the development history of various acoustic transmitters.

### 2.2 Why acoustic telemetry? Advantages over other tracking methods

Several technologies are available for tracking animal movement, including radio, satellite, and acoustic telemetry, each with distinct operational characteristics and limitations. Radio telemetry relies on radio-frequency signals transmitted from tags attached to study animals or fish to land-based or aerial receivers. This method is widely used for terrestrial animals and is particularly suitable for freshwater species in rivers, lakes, and coastal areas, where radio signals can travel well. However, radio signals attenuate rapidly in seawater, which severely restricts their applicability in most marine environments [58]. While satellite telemetry uses satellite-connected tags that transmit location and, in some cases, environmental data to orbiting satellites, enabling global tracking of animal movements. This approach enables large-scale tracking of highly mobile species, such as sharks, billfishes, tunas, marine mammals, and sea turtles [56], which partly explains discrepancies when comparing different animal groups. Despite its global coverage, satellite telemetry typically provides lower spatial and temporal resolution, incurs higher costs, and requires larger tags, which limit its use for small-bodied species and fine-scale behavioral studies in aquatic environments [92].

In contrast, acoustic telemetry uses underwater sound waves to monitor aquatic animals within predefined receiver arrays, making it particularly well-suited for submerged and low-visibility environments. Transmitters (tags) emit uniquely coded acoustic signals, which are detected by hydrophone receivers. This method is highly effective for tracking aquatic species, providing fine-scale spatial and temporal data within established receiver arrays. It is cost-effective for large sample sizes when arrays are already established and popular in aquatic movement ecology research. Compared with alternative tracking methods, acoustic telemetry offers several key advantages under appropriate deployment conditions:

• *Higher spatio-temporal resolution:* Acoustic telemetry can provide fine-scale spatial and temporal data within receiver arrays, allowing detailed analyses of movement patterns and habitat use that are often not achievable with satellite-based systems [92,93].
• *Suitability for aquatic environments:* Unlike radio telemetry, which is limited by water’s absorption of radio waves, acoustic signals propagate efficiently underwater. This efficiency makes acoustic telemetry the favored approach for tracking aquatic species across a wide range of depths and habitats [94].
• *Reduced detection bias in localized arrays:* Well-designed receiver arrays can minimize detection gaps and improve estimates of residency and site fidelity, particularly in comparison with broader-scale satellite telemetry approaches [95].
• *Potential for real-time monitoring:* Acoustic telemetry enables real-time tracking of fish movements, providing immediate data on movement and behavior of tagged individuals to support rapid response to ecological changes and conservation needs [96].
• *Minimal impact on animal behavior:* Acoustic tags are typically designed to be compact and lightweight, which minimizes their impact on tagged organisms’ behavior, which is vital for collecting accurate data without disturbing fish’s movements [17,22].

## 3 Acoustic telemetry system architectures and tag design

Building on the advantages of acoustic telemetry discussed earlier, understanding the system’s architecture is crucial for interpreting tracking data and selecting appropriate technologies for specific ecological applications. An acoustic telemetry system generally consists of animal-borne tags, receiver arrays, and supporting communication and data-processing infrastructure. The system’s performance is significantly affected by tag design, power limitations, and sensing capabilities. Within this system-level framework, acoustic tags can be categorized into three main types based on their communication functionalities and additional features: transmitters, transceivers, and sensor-integrated tags. Table 2 compares these categories by highlighting their core functions, typical applications, and associated trade-offs concerning power consumption, battery longevity, data management, advantages, and limitations. This comparison is intended to facilitate an informed selection of tag types for ecological research. In addition, this section examines key technical specifications, operational ranges, and the effectiveness of various tag designs across diverse marine species and environmental conditions.

**Table 2:** **Comparison of acoustic telemetry types**.

Feature	Transmitter tags	Transceiver tags	Sensor-integrated tags
Primary function	Emit coded acoustic signals at preset intervals	Allow communication both from the tag to the receiver and from the receiver to the tag	Combine position signals with environmental/physiological sensors
Communication	One-way (Tag → Receiver)	Two-way (Tag ↔ Receiver)	Primarily one-way (Tag → Receiver); Some advanced models can be bidirectional
Applications	• General movement, habitat use, migration paths • Estimation of survival, dispersal, and residency • Track and monitor the movement of various small fish species (e.g., juvenile salmon [85], juvenile sturgeon [86], juvenile eels and lampreys [88], etc.)	• Enabling real-time performance tracking [100] • More accurate tracking of animals, monitoring behaviors, and collecting environmental data [101] • Adaptive monitoring in dynamic environments • Remote adjustment of tag settings (ping rate, sensor activation)	• Biologging: Depth, temperature, acceleration (behavior), heart rate (physiology) • Predation detection: e.g., sudden temperature or pH change • Fish farming and seaweed monitoring [12] • Monitor physical activity and respiratory frequency [11]
Power source	Primary micro battery (e.g., lithium/carbon fluoride (Li/CFx) battery)	Power system (batteries and magnetic power switch)	Primary cell battery (higher drain from sensors, e.g., LiPo battery)
Battery life	Longest (months to years)	Moderate (receiver function consumes power)	Shortest (sensors and processing consume significant power)
Data retrieval	Passive: Data are only retrieved when the tag is within range of a receiver	Interactive: Data can be requested, and tag settings can be changed remotely	Passive (standard) or Interactive (advanced models)
Examples of tags	JSATS [85], lamprey/eel tag [88], AMT tag [17]	Tags used in [[102], [103], [104]]	IMPAQT [105], AE-FishBIT [11]
Advantages	• Small size (suitable for small species) • Low cost • Longer operational lifespan • Long deployment periods • Eliminating the need for surgical implantation and suturing • Improve detection range	• Real-time adaptability • Reduced energy waste • Remote configurability and advanced signal processing capabilities • Adjust study design post-deployment • Receivers can send queries or commands • Tags can adapt transmission based on external input	• Multifunctional data (monitoring, data analysis, and decision making) • Multi-purpose (gather and transmit sensor data in inland, coastal, and offshore) • Ultra-low-power, implantable biosensor [11] • Correlate movement with physiology/environment.
Limitations	• No feedback from receivers • Collect data only when within detection range • Tags cannot be reconfigured after deployment • No data if animal leaves the array • Fixed transmission schedule	• Larger size due to added circuitry • Higher energy demand • More complex receiver infrastructure • Shorter battery life than the unidirectional tag	• Shortest battery life • Largest physical size • Data volume requires robust processing

### 3.1 Transmitter-based acoustic tags

Transmitter-based acoustic tags are the most common and fundamental type of acoustic tag used in aquatic telemetry. They are simple, battery-powered devices that emit a unique acoustic signal, or “ping,” at regular intervals. Their primary purpose is to broadcast their identity or basic data, like sensor readings, into the environment. They lack the ability to receive or process signals from other devices. This simplicity leads to smaller size, extended battery life, and lower cost compared to more complex systems. As a result, they are used to track and monitor fish movements and migration patterns in dams, rivers, fisheries, cages, and aquaculture systems. Conventional tags typically need surgical implantation or external attachment.

Several commercial transmitter tags illustrate the classic trade-offs among size, signal strength, and operational lifespan. For example, the Sonotronics PT-1 transmitter [97] has a cylindrical shape (7.1 mm diameter, 16.0 mm length; 1.25 g dry weight), with a detection range of 300 m, but its short operational lifespan of only 7 days at a 1-s PRI (pulse repetition interval) limits its suitability for long-term studies. The HTI 795LG transmitter [98] provides improved durability, operating for approximately 220 days at a 3-s PRI or 400 days at a 10-s PRI, but is larger size (11 mm × 25 mm) and heavier (4.6 g), along with a lower signal strength of 152 dB, limiting its use with smaller species. The Vemco V7 transmitter [99], smaller (7 mm × 18 mm), lighter (1.4 g), with a comparable lifespan of 220 days, but requiring a slower transmission rate (90-s PRI), and has a lower output (136 dB), limiting its detection range. Collectively, these constraints often restrict transmitter-based studies to larger fish and introduce physiological and behavioral biases in smaller species.

A significant advancement in transmitter design was achieved by Deng et al. [85] with the development of the Juvenile Salmon Acoustic Telemetry System (JSATS), the first injectable acoustic fish transmitter that eliminates the need for surgical implantation. This reduced costs, tag burdens, handling time, and adverse biological effects, enabling studies on previously untaggable species and size classes. By enabling safer, more accessible tracking, it provides vital insights for salmon recovery and the design of fish-friendly hydroelectric systems. The JSATS transmitter integrates three primary components: a lead zirconate titanate (PZT) tube transducer for acoustic emission, a compact electronic control circuit, and a lithium/carbon fluoride (Li/CFx) battery. Two variants of JSATS, V1 and V2, share the same size, weight, and form factor but differ in performance. V1 offers adjustable source levels up to 158 dB (re 1 μPa at 1 m), supporting high-resolution tracking, whereas V2 provides a fixed source level of 155 dB with substantially extended battery life (over 100 days at a 3-s PRI), outperforming V1 and commercial alternatives (which last under 20 days at similar settings). This dual configuration enables flexibility between signal strength and longevity, depending on study objectives.

The JSATS transmitter design (Fig. 3a) comprises three main components housed within an epoxy cylinder. The front section, housing the PZT transducer and circuit board, is smaller in diameter than the rear section to reduce weight and improve acoustic performance. It measures (15.0 mm × 3.38 mm) and weighs 217 mg, allowing implantation with an 8-gauge needle, and is approximately 30% lighter than commercial tags [66], substantially decreasing the tag burden, which is the ratio of transmitter weight to fish weight. They developed a Li/CFx micro-battery measuring (6.0 mm × 3.0 mm × 70 mg) with a jelly roll design, achieving a cylindrical form factor, making it suitable for use in capsule-shaped transmitters. Its modular battery design allows capacity scaling for increased lifespan or higher acoustic output, making it adaptable across species sizes. The PZT-5H ceramic tube transducer operates in hoop mode, emitting omnidirectional sound at 416.7 kHz, consistent with JSATS system requirements.

**Fig. 3:**
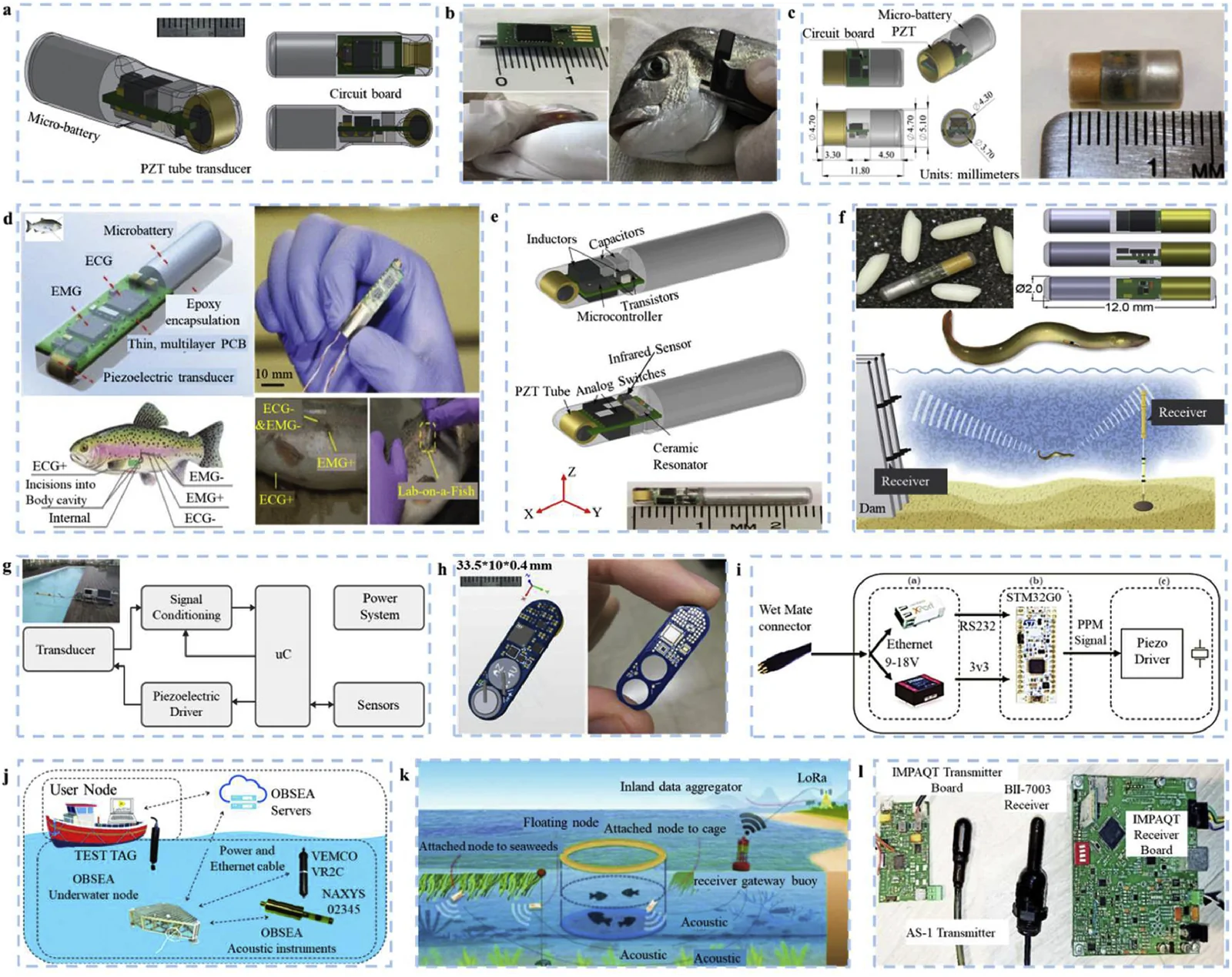
**Different types of acoustic tags.** (a) Injectable JSATS transmitter [85]. Copyright 2015, Springer Nature. (b) Photograph of AE-FishBIT v.1s before and after encapsulation (left) and attached to sea bream operculum (right) [11]. Copyright 2019, Frontiers. (c) Design of JSATS 208.3-kHz acoustic transmitter. Left shows a computer-aided design; right shows the transmitter prototype (smallest ruler division is 1 mm). [22]. Copyright 2023, IEEE. (d) Lab-on-a-Fish enables wireless in vivo monitoring of aquatic animals’ physiology, behavior, and environment. (Top-left) 3D rendering shows the device’s components: a thin, multilayer PCB with sensors, an acoustic transducer, and a microbattery in biocompatible encapsulation. (Top-right) Device photograph. (Bottom-left) Schematic of the tagging protocol showing incision, probe placement, and Lab-on-a-Fish positioning. (Bottom-right) Photos depict the implantation process and device location with ECG and EMG probes [90]. Copyright 2021, IEEE. (e) Design of the long-lifetime acoustic transmitter [86]. Copyright 2016, AIP. (f) The upper left shows the AMT next to a few grains of rice. The upper right shows a computer-assisted design of the AMT. The bottom shows a use case of the acoustic transmitter (eel tracking near a hydroelectric dam) [17]. Copyright 2021, Elsevier. (g) Block diagram of the bidirectional tag hardware. (h) The left is a 3D model of the bidirectional tag, and the right is the PCB manufactured (g and h) [102]. Copyright 2021, IEEE. (i) Systems’ process flow diagram shows three blocks: left is communication and power, center is the microcontroller, and right is the piezoelectric element with its driver [103]. Copyright 2023, SARTI. (j) Testbed framework first approach, iteration over the test tag and instruments installed on the OBSEA underwater cabled observatory [100]. Copyright 2023, IEEE. (k) IMPAQT telemetry platform [105]. Copyright 2020, ThinkMind. (l) IMPAQT ultrasonic transmitter [117]. Copyright 2021, IARIA.

Building on earlier injectable designs (e.g., [41,85]), Lu et al. [86] developed a compact, high-performance transmitter (24.2 mm × 5.0 mm; 0.72 g) addressing the size-longevity trade-off in acoustic telemetry. Operating at 416.7 kHz with adjustable source levels (159–163 dB (re 1 μPa at 1 m)), the transmitter achieves a theoretical detection range of up to 500 m and was designed for 1 year of operation at a 15-s PRI. The system (shown in Fig. 3e) integrates a custom Li/CFx battery, an electronic control board, and an optimized piezoelectric transducer, enabling long-term monitoring of small fish species such as juvenile sturgeon while maintaining detection reliability. The transducer utilizes a Type VI PZT ceramic tube (outer diameter (OD): 2.54 mm, inner diameter (ID): 1.80 mm, length: 2.65 mm) that resonates at 416.7 kHz for JSATS compatibility. A radial offset of 0.15 mm creates a tapered wall, thicker near the circuit board and thinner at the front. This asymmetric design concentrates electric fields in the thinner section, directing most of the acoustic energy forward for precise signal transmission. An 8-bit PIC16LF1823 microcontroller [106] generates waveforms using phase-shift keying (PSK) to transmit a 31-bit tag identifier, including a 7-bit header for synchronization, a 16-bit unique identification number, and an 8-bit cyclic redundancy check for error detection. A 10 MHz ceramic resonator maintained timing accuracy, setting PSK frequency at 416.7 kHz with an accuracy of 0.5% during transmission.

An ultra-lightweight acoustic micro-transmitter (AMT) was developed by Deng et al. [17], weighing 0.08 g, making it one-third lighter than existing options. It achieves a source level of 148 dB, operates for 30 days with transmissions every 5 s, and achieves 100% detection of tagged fish, confirming its effectiveness for tracking early-life-stage species. The AMT gathers data for ecological policy, environmental conservation, and resource management, tracking small aquatic animals, like juvenile American eels and Pacific lampreys, weighing as little as 4 g. The AMT comprises three main components (Fig. 3f): (1) a PZT ceramic tube transducer (3.4 mm in length, 1.8 mm in OD, and 1.4 mm in ID) designed for vibration mode at 416.7 kHz along its axis; (2) an electronic circuit board with a compact EFM8SB10F8G microcontroller (1.78 mm × 1.66 mm) that generates timed, modulated signals, optimizing energy use; (3) a Li/CFx micro-battery (MB204), measuring (1.8 mm × 4.2 mm in length; 20 mg) and a minimal volume of 11 mm³, providing over 3.3 V and a 1.4 mAh capacity for long-term operation, with a detection range of 80–140 m depending on noise.

Overall, transmitter-based acoustic tags remain the fundamental instrument in aquatic biotelemetry. Continuous progress in miniaturization, battery technology, and transducer design persistently broadens their applicability across diverse species sizes, habitats, and research durations, while simultaneously minimizing behavioral bias and ecological impact.

### 3.2 Transceiver-based acoustic tags

While transmitter-based acoustic tags constitute the foundation of most aquatic telemetry studies, their strictly unidirectional communication constrains system adaptability, precise ranging, and network-level intelligence. These limitations have motivated the development of transceiver-based acoustic tags, which facilitate bidirectional communication, real-time interaction, and integration with underwater sensor networks and autonomous platforms. Such systems represent a crucial evolutionary step toward interactive, energy-efficient aquatic monitoring architectures. Transceiver-based acoustic tags integrate both transmission and reception capabilities within a single device, enabling bidirectional communication in aquatic telemetry systems. Unlike conventional transmitter-only tags, transceivers can receive, decode, and respond to acoustic signals from other tags, receivers, or dedicated acoustic gateways. This capability represents a significant advancement in aquatic monitoring, enabling real-time interaction, single-base transceiver ranging, and enhanced behavioral observation of aquatic organisms. These features support more precise movement tracking and provide valuable insights for addressing anthropogenic pressures such as overfishing, habitat fragmentation, and environmental change.

Current acoustic telemetry systems rely on unidirectional communication, meaning tags only transmit signals but cannot receive input. This constraint limits aquatic research by preventing post-deployment reconfiguration, preventing precise time-of-flight (TOF) measurement for accurate range-based tracking, and restricting the development of underwater sensor networks in which tagged animals could function as mobile sensing nodes [107,108]. To address these limitations, Masmitja et al. [102] introduced a bidirectional acoustic tag enabling real-time reconfiguration and accurate TOF distance measurements, thereby improving tracking for autonomous underwater vehicles. By incorporating onboard processing, these tags operate as intelligent network nodes, supporting underwater wireless sensor networks [[109], [110], [111]], and enabling emerging IoT-based aquatic monitoring and swarm-based research approaches [112], representing a significant shift in our approach to studying marine ecosystems.

The bidirectional tag architecture (Fig. 3g) was designed to balance compact size, ultra-low power consumption, and sufficient computational capabilities. Piezoelectric transducers were tested for performance, all fitting within the maximum size limits (10 mm × 33 mm). The system employs an ultra-low-power STM32L432-series microcontroller with an Arm Cortex-M4 core and integrated digital signal processing, enabling bidirectional communication protocols and efficient range estimation.

A wake-on-acoustic architecture enables the tag to remain in low-power sleep mode until triggered by an incoming acoustic signal, thereby significantly extending its operational lifespan. The tag’s power system incorporates a dual zinc-air batteries with 1.4 V, 175 mAh capacity, and a compact (7.9 mm diameter, 3.6 mm height) button design, and a magnetic power-switching mechanism, allowing complete shutdown during storage or transport to preserve battery capacity. Fig. 3h shows the 3D model and prototype of the small and lightweight printed circuit board (PCB) (33.5 mm × 10 mm × 0.4 mm; 0.2 g), which supports practical deployment on aquatic organisms while maintaining system performance.

To ensure compatibility with long-term ocean observatories and real-world monitoring infrastructure, Batet et al. [103] extended this proof-of-concept [102] into a modular, reprogrammable acoustic platform suitable for ecological research. The system architecture (Fig. 3i) integrates communication, power management, and signal generation within a remotely configurable platform compatible with the OBSEA underwater observatory [113]. The data processing core waits 30 s for firmware updates upon startup before executing default routines. Operating at 69 kHz, the system supports continuous transmission, user-defined pulse sequences (50 pulses at maximum power), and adjustable PPM. Field experiments demonstrated a source level of approximately 145 dB (re 1 μPa at 1 m) and detection ranges of up to 220 m using commercial receivers and 340 m when integrated with OBSEA’s seabed-mounted hydrophone array.

Robust validation of transceiver-based telemetry systems is essential for assessing performance under realistic marine conditions. Building on earlier prototypes [102,114], Gerard et al. [100] developed a comprehensive evaluation pipeline combining controlled laboratory testing with in situ deployment at the OBSEA underwater observatory near Barcelona, Spain [113]. Laboratory experiments assessed acoustic output and signal integrity using calibrated hydrophones, while field trials employed both fixed seabed receivers and mobile platforms to assess detection range and communication reliability. The system demonstrated detection ranges approaching 500 m at 69 kHz, comparable to or exceeding commercial solutions, while offering enhanced configurability and real-time monitoring capabilities. The range evaluation testbed used a surface-mounted emitter-only tag on a boat (Fig. 3j). Signal reception involved OBSEA’s seabed NAXYS-Ethernet-02345 hydrophone and a Vemco VR2C-69kHz receiver. Data could be monitored in real-time or analyzed later. The inclusion of software-defined modem functionality further enabled flexible signal processing and multi-layer data verification under varying environmental conditions.

Advances in firmware design have further enhanced the practicality of bidirectional acoustic tags. Ripoll et al. [115] developed a modular, energy-efficient firmware architecture for the SASES hardware platform [114] via an STM32L432KC microcontroller [116], enabling flexible switching between modulation schemes while maintaining system stability. The layered architecture separates low-level (transmission-layer) hardware control from intermediate-level (communication-layer) protocols, simplifying waveform development and facilitating the integration of additional sensors. This design improves scalability and supports long-term deployments without substantially increasing power consumption.

To further overcome the constraints of conventional unidirectional systems, Xaus et al. [104] developed the first comprehensive software framework for bidirectional acoustic communication, representing a major step forward in underwater telemetry system design. This framework employs PPM for robust data encoding and threshold-based demodulation to enhance reliability in challenging aquatic environments. Experimental results demonstrated operational lifetimes of up to 110 days in pinger mode and approximately 72 days during active bidirectional communication, highlighting the feasibility of sustained interactive monitoring.

The integration of bidirectional acoustic telemetry with autonomous underwater vehicles (AUVs) further extends the capabilities of aquatic monitoring systems. Batet et al. [101] developed a framework for reliable bidirectional communication between AUVs and tagged organisms. Experiments show this approach enables real-time data sharing and reliable signals in challenging underwater conditions. The system features protocols for synchronizing AUVs with tags and adaptive navigation, allowing route adjustments based on tag signals. This interaction enhances tracking accuracy and behavioral monitoring while enabling responsive data collection strategies. Such systems open new opportunities for coordinated marine observation, swarm robotics, and ecosystem-scale monitoring, marking a transition from passive tracking to interactive, adaptive aquatic telemetry networks.

### 3.3 Sensor-integrated acoustic tags

While transceiver-based acoustic tags facilitate bidirectional communication and interactive tracking within underwater telemetry networks, recent advancements further extend these capabilities by incorporating miniaturized sensors directly into acoustic tags. Sensor-integrated acoustic tags augment both transmitter- and transceiver-based architectures, enabling the concurrent collection of behavioral, physiological, and environmental data alongside positional information. This development signifies a paradigm shift from solely location-focused tracking toward comprehensive, multi-parameter observation, thereby supporting data-driven research in aquaculture, ecosystem monitoring, and integrated multi-trophic aquaculture (IMTA) systems.

Sensor-integrated acoustic tags are not a mutually exclusive category; rather, they extend conventional transmitters and transceivers by incorporating sensors that monitor physiological and environmental parameters such as temperature, pressure, acceleration, heart rate, and dissolved oxygen. Sensor measurements are either embedded in the acoustic signal or stored on board and transmitted when communication conditions permit. By combining acoustic telemetry with in situ sensing, these systems enable multi-dimensional observation of aquatic organisms and their environments, advancing applications in precision aquaculture, ecosystem monitoring, and ecological research. Precision fish farming (PFF) increasingly relies on sensor-enabled telemetry to optimize production efficiency, animal welfare, and environmental sustainability. Recent advances in miniaturized, low-power sensors allow non-invasive monitoring of marine species in both controlled and natural environments, providing insights into behavior, physiology, and environmental interactions. These capabilities support real-time decision-making in aquaculture and enhance long-term ecological research.

Within this context, the European IMPAQT project focuses on improving IMTA through innovative monitoring technologies. Jafarzadeh et al. [105] introduced a multifunctional underwater acoustic telemetry platform designed for distributed sensing, data analysis, and decision support (Fig. 3k). The system comprises multiple ultrasonic transmitter nodes and a gateway buoy that aggregates sensor data and forwards it to a central system via long-range communication. Each node integrates environmental sensors, including accelerometers, temperature, and pressure sensors, and supports external sensor connections. Designed to be lightweight and compact, the transmitters can be attached to seaweed or deployed as free-floating nodes, minimizing environmental impact while enabling real-time, multi-parameter monitoring in IMTA systems. The original IMPAQT transmitter employed a compact Type VI PZT ceramic transducer and ultra-low-power electronics to balance sensing capability, communication range, and energy efficiency. Powered by a rechargeable LiPo battery with integrated power management, the system achieved operational lifetimes ranging from weeks to several months, depending on sampling and transmission duty cycles. These design choices demonstrate how sensor integration can be achieved without significantly increasing tag size or energy consumption.

Subsequent work simplified the IMPAQT telemetry platform to reduce system complexity, cost, and environmental footprint while preserving core functionality [117]. As shown in Fig. 3l, the revised architecture consists of four main components: a piezoelectric transducer, a transducer driver, a modulation controller, and a boost converter to enhance transmission power. The receiver uses the BII-7003 hydrophone [118], with a broad frequency response (1 Hz–560 kHz), suitable for various transducers and deep aquaculture monitoring. The transmitter uses the AS-1 transducer (Aquarian Scientific) [119] with a wide bandwidth (1 Hz–100 kHz). It has a compact design (12 mm × 40 mm) and a 200 m depth rating. It operates bidirectionally as both a transmitter and a receiver, achieving a source level of 140 dB (re 1 μPa at 1 m) at 90 kHz, matching the IMPAQT platform’s range and data requirements. On-board sensors were removed in favor of external sensor integration via standard wired interfaces, improving flexibility and compatibility across monitoring scenarios. The transmitter tag has a 1200 mAh LiPo battery [120] with integrated power management that supports a lifespan of 2 weeks to 6 months on a single charge. This redesign resulted in extended battery life and improved adaptability for diverse aquaculture deployments while maintaining comparable detection ranges and acoustic performance.

Beyond environmental sensing, sensor-integrated tags have enabled direct physiological monitoring of farmed fish. Within the AQUAEXCEL2020 EU project, Martos-Sitcha et al. [11] developed AE-FishBIT v.1s, an ultra-low-power, implantable biosensor capable of simultaneously measuring physical activity and respiratory frequency, a dual function absent in commercial devices. Validated on Mediterranean species such as European sea bass and gilthead sea bream [121]. The compact system measures (14 mm × 7.2 mm × 7.0 mm; weighs 600 mg), and integrates a tri-axial accelerometer with a ± 8 g range and 800 Hz sampling rate, a high-performance Li-ion battery supporting rapid 120-s charging, and is built on a 0.8 mm four-layer PCB. It features a microprocessor with 256 KB of flash memory and 32 KB of RAM, plus an RFID tagging module for animal identification. The device is waterproof, withstands 6 bars of pressure in air and seawater, and maintains electrical isolation via protected pins, making it suitable for long-term implantation in marine species, as shown in Fig. 3b.

The integration of near-field communication (NFC) technology into miniature data acquisition systems, as demonstrated by Peres et al. [12], tackles monitoring challenges in IMTA. The low-power device includes sensors for temperature, light, depth, and motion, logs data internally, and transmits data wirelessly via NFC to smartphones. It features an onboard microcontroller with machine learning for real-time analysis. The mechanical design includes attachment mechanisms for secure deployment on IMTA organisms: textured surfaces for adhesive bonding to seaweed blades, perforations for safety lines to mooring systems or kelp stipes, and sensors for temperature and depth that maintain water contact for accurate measurements. It supports bidirectional NFC for remote configuration, status checks, and data retrieval, combining sensing with deployment features for dynamic aquaculture environments.

Finally, Table 3 provides a comprehensive comparison of the specifications of miniaturized acoustic transmitters used in marine animal tracking. The table summarizes the key parameters, including dimensions, dry weight, source level (dB), tag lifetime (varying PRI), detection range, and operating frequency. The “None” in the table indicates that it is not mentioned in the study.

**Table 3:** **Specification comparison between different acoustic transmitters. Ø stands for diameter, L for length, and PRI for pulse repetition interval**.

Ref.	Year	Dimensions (mm)	Dry weight (g)	Source level (dB)	Tag lifetime	Detection range	Operating frequency (kHz)
[85]	2015	Ø: 3.38 L: 15	0.217	V1: 158 V2: 155	V1: 20 days at 3-s PRI V2: 100 days at 3-s PRI	Up to 250 m	416.7
[41]	2015	Ø: 3.8 L: 15	0.217	155	130 days at 3-s PRI	None	416.7
[86]	2016	Ø: 5 L: 24.2	0.72	159–163	98 days (163 dB, 5-s PRI) 127 days (161 dB, 5-s PRI) 285 days (163 dB, 15-s PRI) 365 days (161 dB, 15-s PRI)	Up to 500 m (163 dB)	416.7
[97]	2020	Ø: 7.1 L: 16	1.25	129–133	7 days at 1-s PRI	300 m	69 to 83
[98]	2023	Ø: 11 L: 25	4.6	152	220 days at 3-s PRI 400 days at 10-s PRI	None	307
[99]	2016	Ø: 7 L: 18	1.4	136	220 days at 90-s PRI	None	69
[87]	2017	None	0.5	None	13 days at 5-s PRI 25 days at 10-s PRI 48 days at 20-s PRI	None	200
[89]	2020	5.6 × 4.3 × 12.7	0.65	143	65 days at 5-s PRI	150–200 m	180
[88]	2019	Ø: 2 L: 12	0.08	None	30 days	None	416.7
[11]	2019	14 × 7.2 × 7	0.600	None	None	None	416.7
[21]	2020	Ø: 5.1 L: 11.8	0.45	153	140 days at 5-s PRI	250–300 m	208.3
[17]	2021	Ø: 2.0 L: 12.0	0.08	148	30 days at 5-s PRI	80–140 m	416.7
[22]	2023	Ø: 5.1 L: 11.8	0.45	153	15.4 days at 0.5-s PRI 140 days at 5-s PRI	Up to 330 m (270–330)	208.3
[102]	2021	33.5 × 10 × 0.4	0.2	115.5	80 days for Polling-based 370 days for Silent modes 230 days for Mixed mode Tag transmission period 60 s	At least 200 m	50
[23]	2021	15 × 6 × 6	1.1	None	Monitoring behavior for 4 days	None	50
[90]	2021	5.5 × 6.5 × 37	2.4	156.3	280 days	Up to 400 m	416.7

Miniaturization of acoustic tags introduces inherent trade-offs between size, battery life, and system performance. For instance, ultra-lightweight, miniaturized tags (e.g., AMT [17], weighing 0.008 g) are suitable for tagging small species (e.g., early-life-stage species such as juvenile American eels and Pacific lampreys) or for short-term behavioral studies. Their reduced battery capacity significantly limits operational lifespan (30 days at 5-s PRI). This constraint can limit their applicability for long-term monitoring or large-scale migratory studies (such as those involving sharks and salmon smolts), where continuous tracking over extended periods is required. On the other hand, larger tags, such as long-lifetime acoustic transmitters [86], provide longer operational lifetimes (about 1 year at a 15-s PRI), have greater detection ranges (up to 500 m), and support additional sensors, but may impose physiological burdens on smaller organisms. Furthermore, the detection range increases with source level and tag size, further affecting spatial coverage. Consequently, selecting the appropriate tag design requires balancing biological constraints with technical requirements, such as study duration, spatial scale, and data resolution.

## 4 Applications of acoustic telemetry in marine monitoring

Based on the acoustic telemetry system architectures and tag designs discussed in Section 3, this section examines how these technologies are applied in real-world marine monitoring and management contexts. Acoustic telemetry has become a powerful tool for linking animal movement and behavior to environmental pressures and human activities. Here, we highlight its transformative role across four key application areas: assessing the impacts of marine pollution and climate change, supporting the design and evaluation of MPAs, and combating IUU fishing through improved monitoring and enforcement.

### 4.1 Marine pollution impact assessment

As one of the most pressing anthropogenic stressors on aquatic ecosystems, pollution provides a clear example of how acoustic telemetry can bridge the gap between controlled experiments and real-world ecological dynamics. Pollution affects aquatic environments through multiple sources, including agricultural runoff, industrial waste, noise, artificial light, and improper waste disposal [122,123]. Much of the existing research on pollution impacts remains laboratory-based [124,125], limiting its ecological realism and applicability to natural systems [126]. In contrast, acoustic animal tracking enables the collection of behavioral data from free-ranging organisms while simultaneously capturing environmental conditions [127], allowing direct assessment of pollution effects on animal behavior, movement, and welfare in real-world settings [128,129].

Chemical pollutants, such as pesticides, fertilizers, heavy metals, and pharmaceuticals, pose significant risks to aquatic wildlife and human health [130], contributing to biodiversity loss and bioaccumulation even at low concentrations. Acoustic telemetry has been used to investigate the impacts of chemical pollution on various aspects of aquatic life, such as fish mortality [131], species targeted for human consumption [132], and habitat use and avoidance behavior [[133], [134], [135]], thereby supporting ecosystem protection and improving assessments of seafood safety for human consumption. Solid waste disposal represents another major pollution pathway, with marine debris dominated by single-use plastics and industrial waste, including abandoned fishing gear [136,137]. More than 150 million tons of plastic are currently estimated to reside in the ocean, with an additional 4.6–12.7 million tons entering annually [138]. Plastic pollution extends beyond surface waters, accumulating across continental slopes and deep-sea plains [139,140]. Although large-scale removal remains challenging, acoustic animal tracking has proven valuable for identifying debris transport pathways from land to sea and across open-ocean and deep-sea environments [141]. Such insights can inform targeted, localized management strategies with cascading benefits for broader marine ecosystems [142]. Table 4 summarizes key applications of acoustic telemetry in marine pollution assessment, highlighting both its strengths and limitations across different pollution contexts.

**Table 4:** **Advantages and disadvantages of acoustic tracking in marine pollution studies**.

Research focus	Advantages	Disadvantages	Key findings	References
General pollution source	Comprehensive overview of pollution types (chemical, noise, plastic)	Lacks actionable mitigation strategies	Pollution impacts all aquatic ecosystems via diverse anthropogenic sources	[122,123]
Lab vs. field studies	Controlled experiments isolate pollutant effects	Findings may not reflect real-world ecosystem complexity	Lab studies underrepresent natural behavioral responses to pollution	[[124], [125], [126]]
Acoustic tracking methods	Real-time behavioral/environmental data collection in natural habitats	Requires dense receiver arrays; limited to species with high site fidelity	Enables monitoring of chemical pollution impacts (mortality, habitat use)	[[127], [128], [129], [130]]
Chemical pollution impacts	Links contaminants to biodiversity loss and human health risks	Focuses on specific species (e.g., human-consumed fish)	Pollutants (pesticides, heavy metals) alter fish behavior and survival	[[131], [132], [133], [134], [135]]
Plastic pollution	Tracks debris pathways from land to deep-sea environments	Challenges in tracking microplastics and remote/deep-sea accumulation	Over 150 million tons of plastic in oceans; acoustic tracking aids localized cleanup	[[136], [137], [138], [139]]
Management applications	Guides mitigation strategies (e.g., debris removal, fishery regulations)	Limited scalability for global ocean coverage	Passive acoustic systems cost-effectively monitor IUU fishing and debris	[141,142]

### 4.2 Climate change impact assessment

Beyond localized pollution effects, climate change is a global, long-term driver of transformation in marine ecosystems, reshaping species distributions, physiological performance, and ecosystem functioning. Human-induced global change has already had profound impacts on the marine environment [143], with the ocean absorbing approximately 90% of excess heat and 30% of anthropogenic CO_2_, leading to widespread warming and ocean acidification [144]. Rising temperatures and altered circulation patterns have also contributed to declining oxygen levels in both coastal and open-ocean waters [145,146], while global warming has driven many marine species to shift their distributions poleward [147]. The effects of ocean acidification on marine biodiversity remain an area of active debate [148]. Physiological studies indicate that acidification can disrupt sensory systems and increase larval mortality [149,150], whereas some large-scale reviews report limited direct impacts on adult fish populations [151]. These findings emphasize the complexity of climate-induced stressors and underscore the need for comprehensive approaches that integrate physiology, behavior, and ecology across spatial and temporal scales.

Ocean acidification often co-occurs with warming and deoxygenation, underscoring the importance of studying these stressors collectively to support sustainable marine resource management. Biologging tools, particularly acoustic telemetry, play a critical role in this effort by enabling long-term monitoring of animal behavior and physiology in natural environments [129,152]. Sensor-equipped acoustic transmitters measuring temperature, depth, and acceleration allow continuous assessment of habitat conditions and behavioral responses, complementing laboratory-based studies and improving understanding of real-world ecological impacts.

Acoustic tracking has been widely applied to investigate how environmental conditions, especially thermal gradients such as seasonal thermoclines, influence aquatic animal behavior and habitat selection. Many predators exhibit preferences for warmer waters, both regionally and vertically, including common dentex (*Dentex dentex*) [153] and yellowfin tuna (*Thunnus albacares*) [154]. In contrast, several coastal species, such as brown trout (*Salmo trutta*) [155] and Atlantic cod (*Gadus morhua*) [156], preferentially occupy cooler habitats. Acoustic telemetry has also been used to examine responses to oxygen availability; for example, striped bass (*Morone saxatilis*) tolerate a wide temperature range but actively avoid high temperatures and hypoxic conditions during the summer months [157]. Together, these studies provide critical data on species-specific environmental thresholds, offering valuable insights for predicting population responses to ongoing and future climate change. Table 5 summarizes key applications of acoustic telemetry in assessing warming, acidification, and deoxygenation, including methods, advantages, limitations, and major findings.

**Table 5:** **Advantages and disadvantages of acoustic tracking in climate change impact assessment**.

Research focus	Advantages	Disadvantages	Key findings	References
General climate change drivers	Comprehensive analysis of warming, acidification, and deoxygenation impacts	Limited integration of synergistic effects in mitigation strategies	Oceans absorb 90% of excess heat and 30% of CO_2_; species shift poleward	[[143], [144], [145],^147^]
Ocean acidification debate	Highlights methodological discrepancies (lab vs. field studies)	Conflicting conclusions on direct impacts (e.g., sensory vs. population effects)	Acidification disrupts sensory systems [149], but no clear population declines [151]	[[148], [149], [150], [151]]
Synergistic stressors	Emphasizes combined effects (acidification, warming deoxygenation)	Complex data integration required for multi-stressor analysis	Tackling combined stressors is critical for sustainable marine resource use	[129,152]
Thermal preference studies	Reveals species-specific thermal niches (e.g., warm vs. cold-water preferences)	Limited to species with detectable movement patterns	Predators favor warm waters (e.g., tuna [154]); coastal species prefer cold [155,156]	[[153], [154], [155], [156]]
Oxygen concentration effects	Tracks hypoxia avoidance behaviors in dynamic environments	Limited spatial resolution in deep-sea/low-oxygen zones	Striped bass avoid hypoxic zones despite broad thermal tolerance	[157]

### 4.3 Defining marine protected areas (MPAs)

While pollution and climate change assessments focus on understanding environmental impacts, effective marine conservation ultimately requires translating ecological knowledge into spatial management strategies. Over the past 3 decades, MPAs have emerged as a central tool for safeguarding, conserving, and restoring marine species and ecosystems, receiving broad support from the scientific community [158,159]. In alignment with United Nations Sustainable Development Goals 14 (SDG 14), a global target was set to fully protect at least 10% of coastal and marine areas by 2020. However, assessments by UNEP-WCMC and IUCN indicate that only 7.65% of marine areas were protected as of 2021 [160]. Although MPAs are widely recognized for their contributions to biodiversity conservation, fisheries management [161], and climate change mitigation [162]. Their effectiveness is often constrained by critical knowledge gaps. In practice, MPA design and implementation frequently lack spatially explicit ecological models and locally derived movement data [[163], [164], [165]]. Recent advances in acoustic telemetry have addressed this limitation by enabling long-term tracking of a wider range of marine organisms, including smaller species and early life stages [166]. These technological developments provide high-resolution data essential for evidence-based MPA design and adaptive management.

Numerous studies emphasize the importance of spatial data, such as site fidelity, home range size, and movement patterns, for effective MPA design and management [165,167,168]. Acoustic tracking enables direct estimation of species-specific home ranges, informing minimum MPA dimensions required for effective protection [169]. When integrated into multi-species models, acoustic telemetry data allow assessment of MPA performance across taxa with contrasting movement behaviors [168]. Additionally, movement analyses can identify natural barriers and corridors, offering ecologically meaningful boundaries for MPA networks.

Beyond spatial extent, acoustic telemetry provides insights into temporal activity patterns, including diel, seasonal, and annual cycles. Tracking studies identify migration timing, aggregation periods, migration corridors, and connectivity distances for both juvenile and adult life stages, which is critical for designing coherent MPA networks [170]. Although dispersal during early propagule stages plays a major role in population connectivity for many reef species, understanding post-settlement and adult movement is equally important for evaluating spillover effects and maximizing conservation benefits. When combined with abundance and biomass data from experimental fishing or underwater visual surveys, acoustic telemetry supports rigorous evaluation of MPA effectiveness and guides adaptive management strategies [168,171]. Table 6 summarizes the role of acoustic tracking in MPA design and implementation, highlighting its applications in spatial planning, multi-species modeling, and adaptive management, as well as its advancements and limitations.

**Table 6:** **Applications and limitations of acoustic tracking in MPA design and management**.

Research focus	Advantages	Disadvantages	Key findings	References
MPA establishment and SDG14 Goals	Highlights global progress and gaps in marine conservation targets	Limited political/economic commitment; only 7.65% of oceans protected by 2020	SDG14 aims to protect 10% of marine areas by 2020, but progress is insufficient	[[158], [159], [160]]
Challenges in MPA design	Identifies gaps in ecological knowledge and implementation practices	Overlooks local data and spatially explicit models in MPA planning	MPAs often lack integration of empirical data, reducing effectiveness	[[163], [164], [165]]
Acoustic tracking advancements	Enables long-term monitoring of small species and early life stages	Requires dense receiver arrays and species-specific fidelity	Acoustic tracking provides long-term movement data for diverse species (e.g., reef fish)	[166]
Spatial data applications	Informs MPA size, connectivity, and natural boundaries via movement patterns	Limited by species-specific behavior (e.g., migratory vs. sedentary)	Home range estimates guide MPA size [169]; movement barriers define networks	[165,[167], [168], [169]]
Multi-species MPA models	Enhances network design by integrating multi-species habitat use	Complexity in balancing diverse ecological needs	Multi-species tracking improves spillover predictions and MPA efficacy	[168,170]
Activity patterns & connectivity	Reveals diel/seasonal migrations and connectivity for MPA networks	Limited resolution for rare or deep-sea species	Acoustic tracking identifies migration corridors and aggregation sites (e.g., reef sharks)	[170,171]
Integration with other data	Combines tracking with abundance/biomass data for adaptive management	Requires coordination across methodologies (e.g., visual surveys, fishing data)	Synergy with fisheries data improves MPA monitoring and policy	[171]

Acoustic telemetry has played a direct role in shaping real-world MPA design and policy decisions. For instance, in Seychelles, telemetry studies on reef sharks and marine turtles showed that current MPA boundaries did not adequately cover essential habitat-use areas and movement corridors. Based on these findings, the MPA boundaries were redefined, resulting in an approximately 34% increase in effective habitat protection, a change that was subsequently adopted by policymakers [172]. This example highlights how telemetry-derived spatial data can directly inform evidence-based conservation planning. Similarly, in the U.S. Virgin Islands, Kendall et al. [173] demonstrated that acoustic telemetry can be used to measure fish residence times and habitat connectivity. These findings directly support the assessment and adjustment of marine protected area boundaries and overall habitat management strategies.

### 4.4 Combating illegal, unreported, and unregulated (IUU) fishing

Effective marine management extends beyond conservation planning to include tools that facilitate surveillance and enforcement and ensure regulatory compliance*.* IUU fishing includes activities that violate national or international regulations and contribute to the unsustainable exploitation of marine resources [174,175]. Various types of IUU fishing exist [176], as shown in Fig. 4a. For instance, illegal fishing happens when a state conducts fishing without permission from another state. Unreported fishing involves catches that are not declared to relevant authorities, such as the fisheries ministry. Unregulated fishing is conducted by vessels without nationality or operating under flags not recognized by RFMO (Regional Fisheries Management Organizations). IUU fishing can occur within the exclusive economic zone or RFMO zone [177]. IUU fishing can occur within exclusive economic zones or RFMO-managed areas and represents a major obstacle to achieving SDG 14 and the recovery of sustainable fisheries [178]. However, despite significant investments in monitoring, controlling, and surveilling fisheries to combat IUU fishing, these efforts often fail to achieve the desired results [179]. For instance, in West Africa, one of the regions most affected globally by IUU fishing, only about 0.5% of the estimated $2.3 billion in annual economic losses due to IUU fishing is recovered [180]. Satellite-based surveillance systems, including automatic identification systems (AIS) and vessel monitoring systems (VMS), provide a cost-effective method for monitoring fishing activities [[181], [182], [183]]. However, vessels can disable or manipulate these systems to hide their identity and location from monitoring authorities [178,184].

**Fig. 4:**
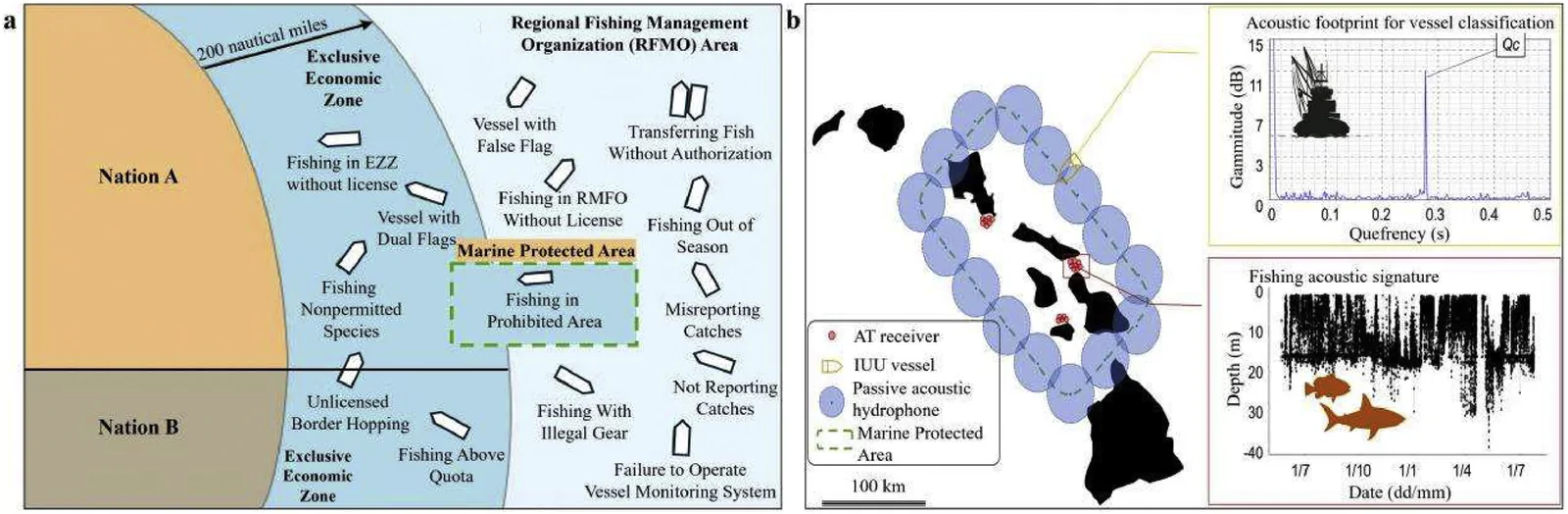
**The concept of illegal, unreported, and unregulated (IUU) fishing.** (a) Common forms of IUU fishing [176]. Copyright 2018, Springer. (b) Schematic of an acoustic surveillance system for monitoring species and vessels at a tropical archipelago with a recently declared MPA. IUU vessel intrusions are detected by a passive hydrophone array (blue points); the blue shadow shows a 12 km detection range [196] deployed along MPA boundaries at the sea floor with a surface antenna/satellite buoy to transmit illegal activity in real-time. The yellow square shows a potential acoustic footprint, detected and classified as a big vessel (from Pollara et al. [194]). The peaks related to engine cylinder rates are labeled Qc. Several coral reefs are monitored by dense receiver arrays with overlapping detection ranges (small red points with a 1 km detection range highlighted by the red shadow). One of these arrays could detect illegal harvests of target species (e.g., groupers or sharks). The red square shows a fishing acoustic signature detected by receivers (from Villegas-Rios et al. [187]) [4]. Copyright 2022, Wiley.

Acoustic tracking offers a complementary approach for detecting and combating IUU fishing by monitoring the movements and survival of aquatic animals. Changes in detection patterns, such as sudden disappearance or abnormal movement behavior, from tagged individuals can be used to infer mortality events [24,73,185,186]. Importantly, recent studies demonstrate that acoustic telemetry can distinguish between fishing-induced mortality and natural mortality, providing a powerful indirect indicator of illegal fishing activity within monitored areas [187].

Acoustic telemetry has been used in real-world case studies to demonstrate its effectiveness in management and policy decisions. In addition to detecting indirect mortality, it has helped identify spatial patterns associated with illegal fishing in large marine protected areas. For instance, in the British Indian Ocean Territory MPA, acoustic tracking of reef sharks showed movement patterns and sudden signal loss in areas linked to suspected fishing activity [188]. These results provided important insights into high-risk zones and enforcement gaps, supporting the development of more targeted surveillance strategies.

To identify IUU fishing using acoustic telemetry, it is necessary to monitor the area with a dense, overlapping array of receivers. The species being tracked should exhibit high site fidelity or a defined home range [187,189]. Acoustic tracking monitoring systems are crucial for identifying IUU fishing activities, as seen in Fig. 4b. They detect suspicious vessels using passive noise recorders, enhancing enforcement efforts and protecting marine ecosystems. Recent advancements in technology, such as the portable noise recorder system [190] and the hydrophone buoy [191], enable detection, classification, and tracking of vessels by analyzing their noise signatures [192,193], as shown in the yellow square in Fig. 4b. These signatures arise from vessel features, including engine size and propeller cavitation [194,195], allowing noise-based classification of vessels. Passive acoustic monitoring provides a cost-effective and discreet means of surveilling large marine areas, with detection ranges up to 24 km in open waters. These systems operate independently and can transmit data in real-time, making them useful for discreetly monitoring IUU fishing activities. When combined with active acoustic telemetry used for marine biodiversity monitoring, passive acoustic approaches significantly enhance situational awareness and enforcement capacity [196]. Table 7 summarizes the applications, advantages, and limitations of acoustic tracking for combating IUU fishing, highlighting key research findings and practical constraints.

**Table 7:** **Applications and limitations of acoustic tracking in combating IUU fishing**.

Research focus	Advantages	Disadvantages	Key findings	References
IUU fishing overview	Highlights global economic losses and regulatory gaps	Limited recovery of losses (e.g., 0.5% in West Africa)	IUU fishing causes $2.3 billion annual losses in West Africa; critical for SDG14	[174,175,178,180]
Surveillance challenges	Exposes limitations of satellite systems (AIS/VMS)	Vessels can disable or manipulate tracking systems	Satellite tools (AIS/VMS) are cost-effective but vulnerable to tampering	[[181], [182], [183], [184]]
Acoustic mortality detection	Distinguishes fishing vs. natural mortality via tagged species patterns	Requires species with high site fidelity or defined home ranges	Mortality spikes in tagged fish indicate IUU activity (e.g., reef sharks)	[24,73,[185], [186], [187]]
Technological solutions	Passive noise recorders detect vessel signatures (engine noise, cavitation)	Limited by vessel noise variability and environmental interference	Hydrophone buoys and portable systems classify vessels via noise	[[190], [191], [192], [193]]
Operational requirements	Dense receiver arrays improve detection accuracy	High initial setup costs; species-specific constraints	Overlapping detection ranges (24 km) enhance monitoring	[187,189,196]
Cost-effectiveness & coverage	Passive systems cover vast areas economically	Limited resolution for small/stealth vessels	Real-time data transmission enables covert monitoring	[190,193,196]

Environmental conditions significantly influence the performance and design of acoustic telemetry systems. In shallow, complex habitats like coral reefs or coastal zones, there is high ambient noise, multipath propagation, signal attenuation, and physical obstructions that require robust modulation techniques, such as BFSK or spread-spectrum methods, along with dense receiver arrays for reliable detection [6,75,151,169]. Conversely, deep-sea environments offer reduced noise and stable signal propagation, enabling longer detection ranges but requiring consideration of pressure resistance and energy conservation [113]. Similarly, noisy environments such as ports or industrial coasts require noise-resistant communication methods and adaptive filtering [197]. These scenario-specific factors are essential for optimizing telemetry effectiveness and ensuring reliable long-term monitoring.

The selection of acoustic telemetry technology depends on specific monitoring objectives and environmental conditions. For example, simple transmitter tags are ideal for large-scale migration studies that require long battery life [56,86], while transceiver systems offer high-resolution positioning and interactive tracking in localized areas [102,113]. Sensor-integrated tags are useful for collecting physiological or environmental data alongside movement information [11,117]. Moreover, energy-harvesting solutions such as wave-powered systems are suitable for long-term deployments in remote offshore environments, whereas batteries are more appropriate for short-term or smaller projects. This scenario-based strategy facilitates more effective planning and deployment of acoustic telemetry systems. To support practical application, a scenario-based decision-making framework is illustrated in Table 8. This framework links monitoring objectives and environmental factors to suitable system configurations, including tag type, communication method, energy strategy, and deployment plan. It is supported by case studies from the existing literature, providing an evidence-based approach to selecting the most appropriate acoustic telemetry solutions across various marine environments.

**Table 8:** **A decision framework based on scenarios to guide the selection of the appropriate acoustic telemetry system configurations, supported by real-world examples**.

Monitoring scenario	Primary objective	Preferred tag type	Modulation scheme	Energy strategy	Deployment strategy	Key trade-offs	Representative case studies/References
Large-scale migration (open ocean)	Long-term movement tracking	Transmitter	PPM, BFSK	Battery (low PRI), Emerging wave energy	Sparse receiver arrays/mobile receivers	Long lifespan vs. low data resolution	Tracking large migratory species like reef sharks [170,172], marine mammals, sea turtles [9,56], and tuna (*Thunnus albacares*) [154]
High-resolution behavioral studies (coastal/reef)	Fine-scale positioning & behavior	Transceiver	CDMA, BPSK	Battery (moderate)	Dense receiver arrays (high overlap)	Accuracy vs. energy consumption	Reef fish residency studies [151,169]; Fine-scale behavioral studies in the aquatic environment [6,75,92]
Physiological & environmental monitoring; Aquaculture monitoring/precision fish farming (PFF)	Multi-parameter sensing (depth, temperature, activity, heart rate)	Sensor-integrated tag	PPM, hybrid schemes	Battery + energy harvesting	Fixed receivers + periodic data retrieval	Sensing capability vs. battery life	IMTA/IMPAQT platform [105]; aquaculture monitoring systems [117]; monitoring physiological parameters like breathing rates in farmed fish [11]; environmental interactions in seaweed habitats [12]
Deep-sea monitoring (>1000 m)	Long-term observations in stable environments	Transmitter/Sensor-integrated	Low-frequency robust schemes	Hybrid (battery + energy harvesting)	Pressure resistant seabed systems	Durability vs. system complexity	OBSEA observatory deployments [113]; deep-sea current measurements [40]
MPA design & conservation	Home range, connectivity, habitat use, site fidelity	Transmitter/Sensor-integrated	PPM (efficient long-term)	Optimized battery (long-term)	Habitat-focused arrays	Coverage vs. cost	Redefining MPA boundaries [172]; MPA design and management (e.g., home range studies, and site fidelity [165,168])
IUU fishing detection & surveillance	Mortality detection (fishing mortality and natural mortality) & vessel interaction	Transmitter/passive acoustic systems	Event-based signaling/coded	Long-life battery/hybrid	Wide-area coverage (dense and overlapping receivers) + noise sensors	Detection reliability vs. infrastructure cost	Mortality inference & predation detection studies [24,73,185,186]; passive acoustic vessel detection (vessel monitoring systems (VMS)) [[181], [182], [183]]
Autonomous long-term monitoring	Multi-year, maintenance-free operation	Transceiver/Sensor-integrated	Adaptive/low-duty-cycle schemes	Battery, Wave energy (TENG/PENG)	Autonomous buoys/seabed nodes	Autonomy vs. system complexity	Wave-powered telemetry systems [50,198]; TENG-based ocean sensors [31,32]; long-term monitoring of small fish species like juvenile sturgeon [86]

## 5 Challenges and limitations of acoustic telemetry systems

While acoustic telemetry has proven its effectiveness in ecological, conservation, and management applications, it faces a range of technical, biological, and operational challenges that can affect data quality, animal welfare, and the interpretation of results. Recognizing these limitations is crucial for designing effective studies and for critically evaluating telemetry-derived data in marine and freshwater environments. The key challenges associated with acoustic telemetry systems include signal attenuation, environmental interference, tag retention issues, and battery life limitations [17,98,[199], [200], [201], [202], [203]], which summarized in Fig. 2b and discussed below. Many of these limitations emerge directly from the system architecture and deployment methods discussed earlier in Section 3.

### 5.1 Signal attenuation and environmental interference

a) *Signal loss and attenuation:* acoustic signal propagation can be affected by environmental conditions such as water depth, temperature gradients, salinity, ambient noise, and physical obstacles like rocks, vegetation, or artificial structures. In complex or cluttered habitats, these factors can reduce detection ranges by up to 50%, resulting in spatial gaps and uneven detection probability [203].
  Marine acoustic telemetry presents additional engineering challenges compared to freshwater systems due to higher salinity, increased ambient noise, and greater signal attenuation, necessitating operation at lower frequencies (usually ≤200 kHz) to achieve adequate detection ranges. Despite these requirements, commercially available miniature marine transmitters remain constrained by limited lifespans, detection ranges, and tag burdens. For instance, Lotek’s Mini MAP M-416 (which weighs at least 0.5 g, lasts 13 days on standard 5-s PRI) [87] and VEMCO’s V5–1H (which weighs a minimum of 0.65 g, lasts up to 65 days, with a maximum source level of 143 dB and a detection range of only 150–200 m under typical ocean conditions) [89] still impose considerable operational constraints. These technical drawbacks present significant barriers to research. The weight limitations mean only larger marine organisms can be tagged, while the short operational life requires frequent recaptures, disrupting long-term behavioral studies. Additionally, the limited detection range limits spatial coverage in open-ocean areas, potentially leading to gaps in data on animal movements.
  To address these limitations, Li et al. [21] developed a miniaturized marine transmitter operating at 208.3 kHz, which outperforms commercial options by providing three enhancements: a reduced weight of 0.45 g, an operational lifespan of 140 days at a 5-s PRI, and an extended detection range from 250 m to 300 m. It has a compact cylindrical design (5.1 mm × 11.8 mm) that integrates a Type-VI PZT ceramic tube transducer (4.3 mm OD, 3.7 mm ID, 3.3 mm length), an electronic control board, and a Li/CFx micro-battery design as [86] with a capacity of 15 mAh and an output voltage of 3.0 V during transmitter operation, optimized for efficient power use and signal propagation in marine environments. Subsequent refinements by Li et al. [22] further improved the detection range to 330 m, representing a 65% improvement over similar devices while maintaining similar size and longevity, demonstrating the feasibility of high-performance, miniaturized marine transmitters. The transmitter design was shown in Fig. 3c. An 8-bit PIC16LF1823 microcontroller and a 10 MHz ceramic resonator generate accurately timed 208.3 kHz square waves to excite the PZT, producing 3 V, 180° phase-shifted waves across the PZT tube to generate acoustic emissions. A 150 μH inductor achieves resonance at 208.3 kHz, enhancing efficiency.
b) *Multipath interference*: reflections from the seafloor, water surface, and submerged structures can generate multipath signals, making accurate localization and time-of-arrival estimation more challenging. Advanced signal processing approaches, including spread-spectrum techniques and improved decoding algorithms, are increasingly used to mitigate these effects, although residual uncertainty may persist under highly reflective conditions [199].

### 5.2 Device and biological limitations

a) *Tag retention and expulsion:* implanted acoustic transmitters may be expelled by the animal or cause physiological stress, which may impact the animal’s health and affect tracking accuracy. For instance, tag expulsion has been observed through surgical incisions. However, in some species, overall survival and growth rates may remain unaffected, highlighting strong species and context-specific responses [200].
  Building on advances in miniaturized acoustic telemetry, Mueller et al. [88] developed a new micro-transmitter for tracking juvenile eels and lampreys, challenging species due to their unique morphology and small size. The tag was manufactured at the Bio-Acoustics & Flow Laboratory at PNNL [204], featuring an ultra-compact design (12 mm × 2 mm; 0.08 g) that enables sutureless implantation through minimal incisions (< 3 mm), improving procedure times, faster healing, and reducing infection risk [205]. The biological impacts of tagging vary by species characteristics, life stage, study duration, anatomical dimensions, environmental conditions, and incision site [16]. These tags emit coded signals at 416.7 kHz, detectable by fixed hydrophone arrays and mobile stations. Their low weight reduces tag burden and biological impact. Along with improved anesthetic and handling techniques, this reduces negative effects on retention, survival, growth, swimming, and predator avoidance [206,207]. Laboratory tests showed no significant impact on swimming or long-term survival in juvenile American eels, with minor expulsion over 30 days. Overall, this micro-transmitter advances aquatic biotelemetry, enabling studies of the movement and behavior of small species while prioritizing animal welfare.
b) *Effects on animal behavior:* the impact of tag size, weight, and implantation method on animal behavior is significant. If tags are not properly miniaturized or implanted, they can alter animals’ natural behaviors, potentially leading to biased study results. Tags that exceed 2–5% of body mass, as recommended by the American Fisheries Society, can hinder swimming performance or predator avoidance, leading to distorted movement data. Miniaturization efforts, such as using tags weighing < 0.5 g for small fish, help mitigate these issues [17]. Tagging small fish species was limited by size constraints until the development of Li et al. [41], which designed a compact, injectable transmitter suitable for implantation via an 8-gauge needle without surgery or suturing. This enabled tagging of smaller-bodied fish and early life stages, reducing size-related research bias. The transmitter includes a piezoelectric transducer, control circuitry, and a battery, achieving an average source level of 155 dB (re 1 μPa at 1 m) and an operational lifespan of 130 days at 3-s PRI, measuring (15.0 mm × 3.88 mm; 217 mg). Its semi-cylindrical shape is designed to minimize impact, with a tapered front that reduces weight while preserving performance. The transmitter utilized a PZT-5H tube transducer with an OD of 2.54 mm, an ID of 1.75 mm, and a height of 1.75 mm. The transducer’s interior cavity was designed to be filled with air. It operated at its resonant frequency of 416.7 kHz, using an 8.5 V signal to improve efficiency. Transducer performance was enhanced by increasing the average source level in the 180° forward projection zone, achieving a 1.5 dB boost from a new off-center tube design, an additional 1.3 dB from a rear-mounted reflector, and optimized spacing to reduce beam distortion. These improvements led to a new injectable transmitter that overcomes size limitations while maintaining acoustic quality.
  Recent studies highlight that tag effects extend beyond size and weight, affecting behavior and predation risk, especially in small or sensitive species [17,41]. Tagged individuals may show reduced swimming efficiency, changed activity levels, or poor predator avoidance, which biases ecological interpretations [23,207]. In some cases, tags are transferred to predators during predation, leading to false movement information [[68], [69], [70]]. To mitigate this, miniaturized predation-detection tags have been developed to distinguish natural movement from predation events [67,201]. Although the commonly cited guideline of keeping tag mass under 2% of body weight offers a useful benchmark, recent research suggests this may not always be sufficient. The impact of tags varies significantly by species, life stage, physiological conditions, and environments [11,23]. Therefore, thorough validation prior to deployment, including behavioral monitoring and pilot testing, is crucial to ensure animal welfare and accurate data collection.

### 5.3 Data reliability and operational limits

a) *Detection accuracy:* although modern systems can achieve high accuracy for specific applications (e.g., predation detection or fine-scale positioning), detection efficiency and temporal precision remain sensitive to environmental noise, tag orientation, and retention variability [201].
b) *False detections and data filtering:* automated detection algorithms may generate false alarms, particularly in noisy environments or densely instrumented arrays, which can reduce responsiveness. To enhance the effectiveness of these systems, incorporate manual validation measures and conservative filtering thresholds to ensure that clinically or ecologically significant events are accurately identified, improving overall system reliability [202].
c) *Battery life and deployment duration:* The operational lifespan of acoustic transmitters is primarily limited by battery capacity and power consumption. Depending on ping rate, transmission power, and sensor payload, tag lifespan can range from approximately 20 days to over 1 year. For example, the HTI 795LG transmitter [98] provides improved durability, operating at a 3-s PRI may have an approximate lifespan of 220 days, while extending the PRI to 10-s can increase lifespan to approximately 400 days, illustrating the trade-off between temporal resolution and longevity. Additionally, Lu et al. [86] developed a compact, high-performance transmitter (24.2 mm × 5.0 mm; 0.72 g) addressing the size-longevity trade-off in acoustic telemetry. Operating at 416.7 kHz with adjustable source levels (159–163 dB (re 1 μPa at 1 m)), the transmitter achieves a theoretical detection range of up to 500 m and was designed for 1 year of operation at a 15-s PRI.

## 6 Emerging advances toward sustainable and autonomous acoustic telemetry

Building on the challenges discussed in Section 5, especially those related to power constraints, maintenance, and long-term autonomy, recent advances in marine telemetry increasingly focus on sustainable and self-powered system designs. As marine telemetry evolves, integrating innovative energy solutions has become critical for overcoming the limitations of traditional batteries in underwater environments. This section highlights two emerging directions: (1) flexible energy harvesting and storage systems that enhance durability, adaptability, and sustainability through bio-inspired and nature-inspired designs, and (2) ocean wave energy harvesting approaches that exploit ambient marine energy to support long-term, autonomous telemetry deployments. Together, these advances address energy autonomy, environmental adaptation, and ecological safety, shaping the next generation of marine tracking systems.

### 6.1 Flexible energy harvesting and storage for acoustic telemetry

All acoustic telemetry devices (tags and receivers) depend on reliable energy storage, making power management a central constraint for long-term and large-scale deployments. Inspired by biological systems, recent advances in flexible energy storage and harvesting include self-healing materials analogous to human skin, stretchable and shape-memory architectures, and biodegradable components designed to function under extreme mechanical deformation. In parallel, flexible energy harvesters, such as solar cells, TENGs, and piezoelectric nanogenerators (PENGs), convert ambient mechanical, solar, and thermal energy into usable electrical power, enabling sustainable and autonomous operation*.* The motivation for integrating flexible energy harvesting with acoustic telemetry stems from the vulnerability of current tags and receivers to mechanical damage, pressure, biofouling, and biological interactions in harsh and unpredictable marine environments. Such failures reduce device lifespan, compromise data integrity, and limit deployment scale. Bio-inspired self-healing materials offer a direct solution by enabling devices to autonomously recover from damage, analogous to healing processes observed in marine organisms such as seal skin regeneration or mollusk shell repair. Because self-healing functionality requires sustained energy input, coupling these materials with robust, bio-inspired energy harvesting mechanisms, particularly TENGs and PENGs driven by wave-induced motion, creates a synergistic, maintenance-free system. This integration paves the way for next-generation self-repairing or unsinkable acoustic tags and receivers, substantially expanding the temporal and spatial scope of marine acoustic telemetry.

#### 6.1.1 Bio-inspired self-healing mechanisms for energy harvesting and storage systems

To meet the demand for autonomous, damage-resistant power systems in acoustic telemetry, bio-inspired self-healing mechanisms have attracted considerable interest for energy-harvesting and storage devices. Extensive research has focused on self-healing nanogenerators, supercapacitors, solar cells, and batteries, aiming to restore critical functionalities, including electrical conductivity, electrochemical performance, optical properties, structural integrity, and, in some cases, biological compatibility. As illustrated in Fig. 5k, self-healing strategies are generally classified as extrinsic or intrinsic. Extrinsic mechanisms rely on pre-embedded healing agents that are released upon mechanical damage, such as the flexible perovskite solar cell developed by Chung et al. [208], where microcapsules containing liquid metal re-establish electrical pathways after cracking (Fig. 5a,b). In contrast, intrinsic self-healing depends on reversible interactions within the material itself, often using polymeric or hydrogel networks with dynamic bonds that enable repeated damage-repair cycles. A notable example is the ionic-skin TENG reported by Lee et al. [209], which employs an ionic polymer conductor to achieve energy harvesting performance approximately twelve times higher than that of silver-based collectors, while exhibiting rapid, autonomous, and repeatable self-healing at room temperature (Fig. 5c,d).

**Fig. 5:**
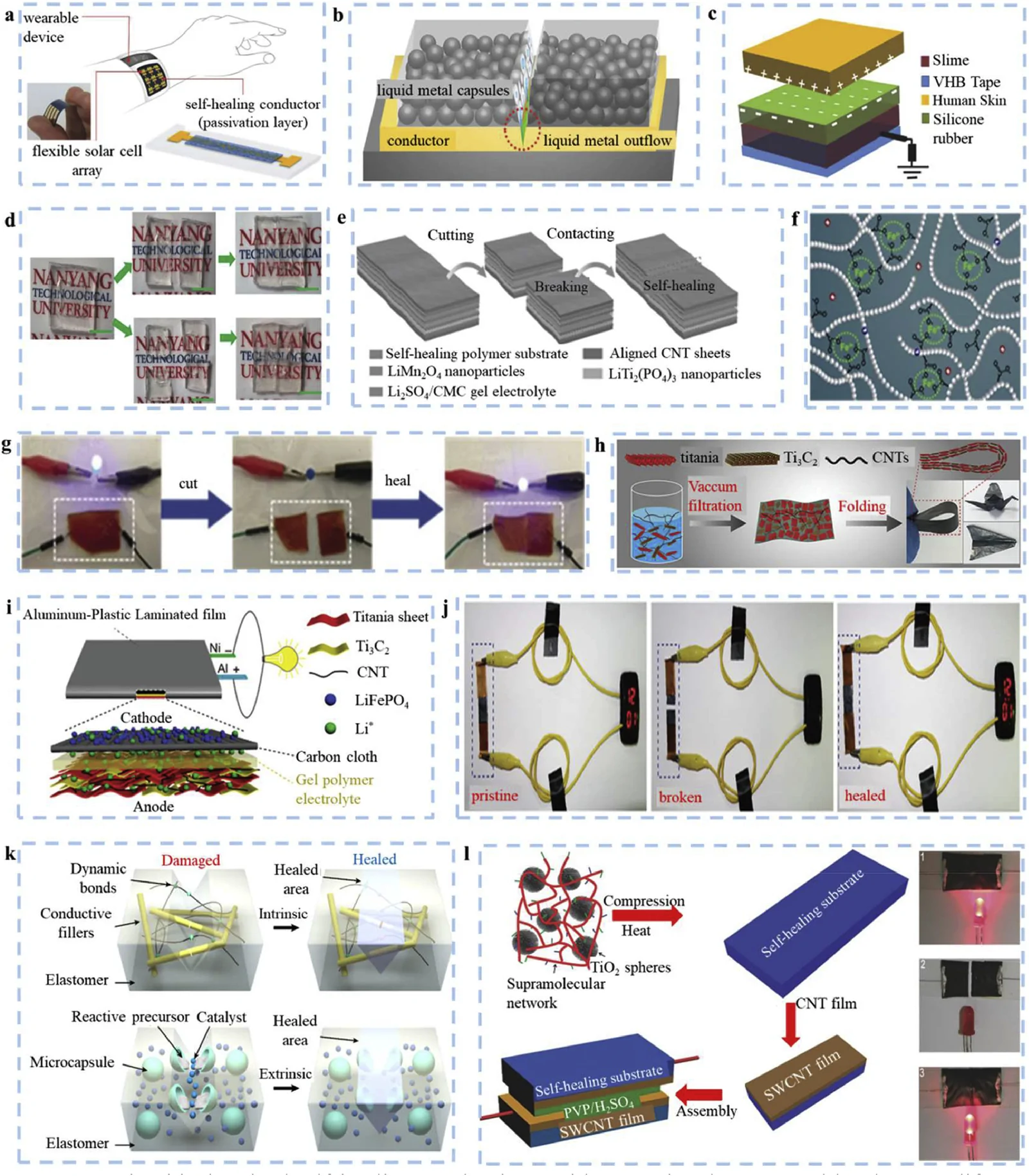
**Integrating bio-inspired self-healing mechanisms with acoustic telemetry tackles damage, lifespan, data integrity, and scalability issues of acoustic tags.** (a) Schematic of a solar-powered smart watch with self-healing conductors. (b) Schematic showing healing of a metal conductor by a passivation film of LMC/polymer composite. Liquid metal flows from ruptured microcapsules to the damaged site. (a and b) [208]. Copyright 2018, Wiley-VCH Verlag GmbH Co. KGaA. (c) Schematic of the IS-TENG. (d) Digital photo of the as-prepared, bifurcated, quadfurcated, and self-healed IS-TENG. (c and d) [209]. Copyright 2017, Wiley-VCH Verlag GmbH Co. KGaA. (e) Schematic of the Li-ion battery’s self-healing process using self-healing substrates [210]. Copyright 2016, Wiley-VCH Verlag GmbH Co. KGaA. (f) Schematic of PANa-Fe^3+^ electrolyte’s self-healability origin. (g) Photographs show the PANa-Fe^3+^ hydro-gel as an ionic conductor connecting the LED circuit before being cut and after healing. (f and g) [211]. Copyright 2018, Wiley-VCH Verlag GmbH Co. KGaA. (h) Schematic showing the fabrication and flexibility of the obtained film, which can be bent or folded into shapes like an “air-plane” and a “crane” without breaking or cracking. (i) Schematic of the flexible battery device using LiFePO_4_ on carbon cloth as the cathode. (j) Photographs of an e-watch powered by the battery before cutting, after cutting, and after self-healing. (h–j) [212]. Copyright 2019, Wiley-VCH Verlag GmbH Co. KGaA. (k) Schematic diagrams of self-healing in compliant conductors: intrinsic self-healing mode (top) and extrinsic self-healing mode (bottom). [213]. Copyright 2024, American Chemical Society. (l) SWCNT films spread on self-healing substrate to assemble self-healing supercapacitors [214]. Copyright 2014, Wiley.

Self-healing flexible batteries present additional challenges due to stringent requirements for ionic conductivity and electrode integrity. Peng et al. [210] reported the first flexible, self-healing aqueous lithium-ion battery (LIB) based on aligned carbon nanotube sheets supporting electrode nanoparticles on a self-healing polymer (Fig. 5e), in which mechanical damage is repaired through an externally triggered healing process mediated by the substrate. In contrast, intrinsic self-healing offers even greater potential for long-term durability. Huang et al. [211] demonstrated an intrinsically self-healing aqueous NiCo//Zn battery using a sodium polyacrylate (PANa) hydrogel electrolyte with ferric-ion-assisted healing (Fig. 5f), enabling autonomous recovery without external intervention. The device retained functionality after repeated damage, successfully powering a blue LED (Fig. 5g) and maintaining over 87% healing efficiency after four cutting–healing cycles. Similarly, Wu et al. [212] developed a self-standing LIB by molecularly coupling 2D titanium oxide and carbide sheets (Fig. 5h), with an intrinsically self-healing poly(ethylene glycol) diamine-based electrolyte (Fig. 5i), allowing the battery to resume operation, powering an electronic watch simply by reconnecting fractured components (Fig. 5j).

Beyond batteries, self-healing supercapacitors have also shown promise for resilient energy storage. Wang et al. [214] reported the first supercapacitor capable of simultaneously restoring mechanical and electrical functionality by coating self-healing polymer substrates with single-walled carbon nanotube (SWCNT) films (Fig. 5l). Upon damage, the composite enables the reconnection of separated SWCNT networks, recovering both structural integrity and charge-storage performance. The device retained up to 85.7% of its original specific capacitance after five successive damage-repair cycles. Overall, these advances highlight the potential of bio-inspired self-healing energy systems to enhance the robustness, longevity, and autonomy of power supplies for next-generation acoustic telemetry devices deployed in harsh marine environments.

#### 6.1.2 Engineered stretchable-compressible energy harvesters and storage: exceeding natural paradigms

While bio-inspired self-healing systems focus on restoring functionality after damage, engineered stretchable and compressible energy devices are designed to sustain uninterrupted operation under extreme, repeated mechanical deformation, often exceeding the capabilities of natural biological systems. The fundamental mechanical properties of human skin, including stretchability and compressibility, have long inspired flexible electronics design [[215], [216], [217]]. However, recent advances in materials science and structural engineering have enabled energy harvesting and storage devices with mechanical resilience and performance that exceed natural paradigms, enabling possibilities for high-performance flexible electronics [[61], [62], [63],[218], [219], [220]]. These developments are particularly relevant for acoustic telemetry devices, which must operate reliably under dynamic mechanical stresses induced by currents, waves, pressure fluctuations, and biofouling in marine environments.

The development of stretchable energy harvesters requires intrinsically stretchable components. In TENGs, mechanical compliance depends on the elasticity of the triboelectric layers and the conductive electrodes. Metallic-based stretchable conductors, including liquid metals, gold nanosheets, and silver nanowires (AgNWs), have emerged as promising solutions due to their high conductivity, mechanical durability, interconnected networks, and ease of fabrication. For example, Lee et al. [221] demonstrated a hyper-stretchable piezo-nanogenerator employing AgNWs as stretchable electrodes, achieving approximately 200% stretchability while maintaining stable energy output (Fig. 6a,b), highlighting the suitability of such architectures for mechanically demanding energy-harvesting applications.

**Fig. 6:**
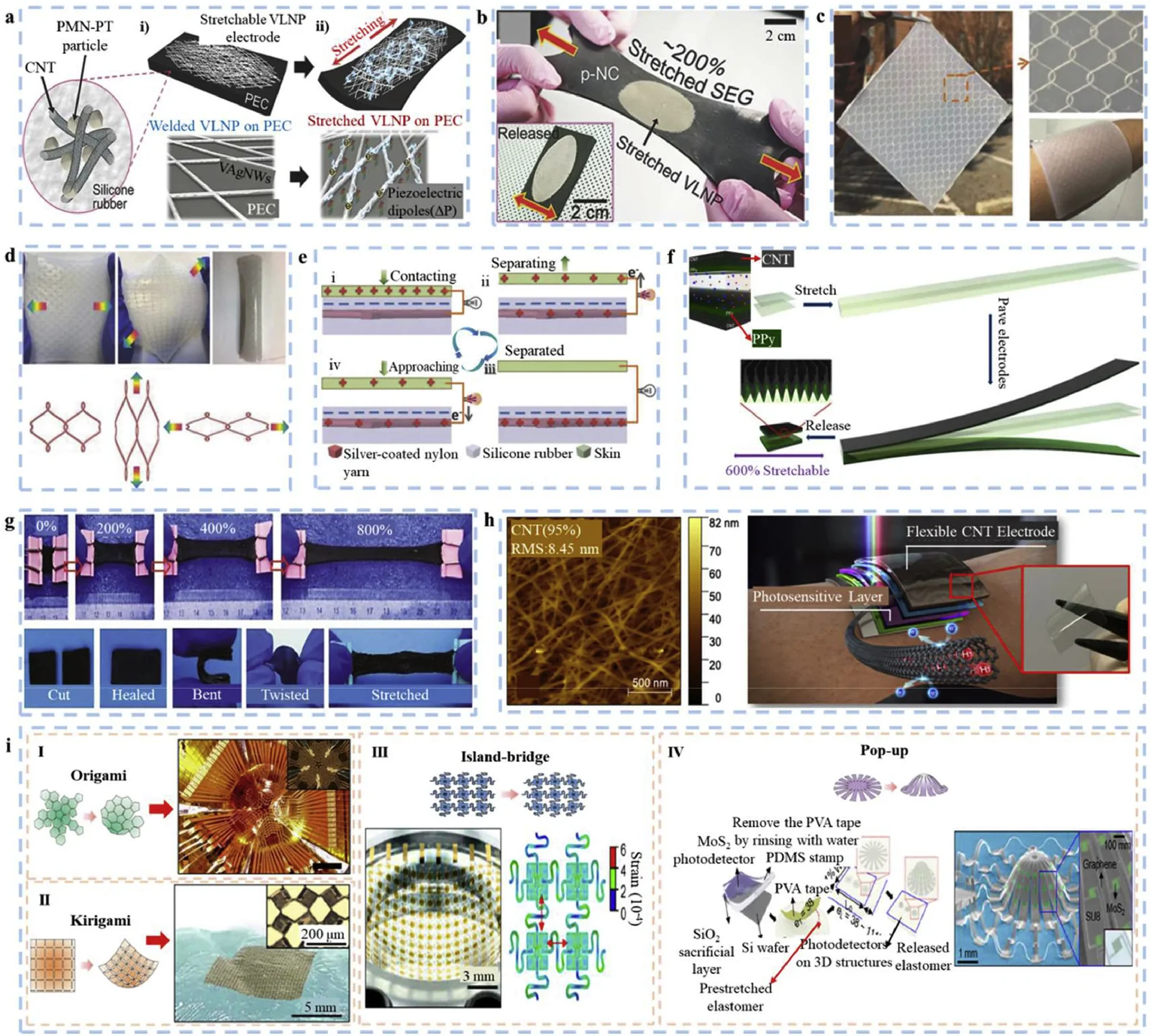
**Integrating stretchable and compressible energy harvesting with acoustic telemetry creates long-term, large-scale, maintenance-free telemetry arrays.** (a) Schematic of a hyper-stretchable, deformable nanocomposite generator. (b) The elastic-composite generator was stretched by hands. The inset shows the SEG is released without damage. (a and b) [221]. Copyright 2015, Wiley-VCH Verlag GmbH Co. KGaA. (c) Photograph of an SI-TENG and its enlarged view, showing it can attach to a person’s forearm. (d) Photograph shows the SI-TENG can be stretched in any in-plane direction and rolled up (top), with a schematic illustrating the in-plane tensile behaviors of the repeated rhombic unit in the SI-TENG system (below). (e) Schematic of SI-TENG operation in single-electrode mode. (c–e) [224]. Copyright 2018, Wiley-VCH Verlag GmbH Co. KGaA. (f) Schematic of the supercapacitor with VSNPs-PAA polyelectrolyte and PPy@CNT paper electrodes (left); and the fabrication of a super-stretchable supercapacitor (right) [225]. Copyright 2015, Springer Nature. (g) Optical images showing the device’s high stretchability (top) and its healing during bending, twisting, and stretching (down) [226]. Copyright 2019, Wiley-VCH Verlag GmbH Co. KGaA. (h) Intrinsically soft, transparent nanocomposite electrodes based on nonmetallic nanostructures. (left) AFM image shows low RRMS of CNT networks; (right) image of the flexible CNT composite electrode [227]. Copyright 2021, Elsevier. (i) Structurally engineered flexible/stretchable PDs. (I) Photograph of a hemispherically concave silicon PD via origami and its flat device before bending (inset) [229]. Copyright 2017, Springer Nature. (II) Photographs of a stretchable kirigami device transferred onto a curvy surface [230]. Copyright 2021, Springer Nature. (III-left) Tilted view of a curved PD array on flexible PDMS membrane. (III-right) Finite element analysis (FEA of a 2 × 2 pixel array at the center of the deformed device shows a 12% biaxial strain, below the fracture range) [231]. Copyright 2011, National Academy of Sciences. (IV-left) Schematic of 3D MoS PD array fabrication via pop-up techniques. (IV-right) SEM images with color highlights of MoS (green), graphene (light gray), and SU-8 (gray). The inset shows a magnified view. FEA results display strain distribution in the device [232]. Copyright 2018, Springer Nature.

Non-metallic ionic conductors based on polymer hydrogels have gained increasing attention as deformable electrodes for flexible energy systems [222,223], featuring exceptional stretchability, optical transparency, self-healing capability, and biocompatibility. When triboelectric materials themselves exhibit limited stretchability or suboptimal triboelectric performance, structural engineering approaches, such as ladder geometries, origami- and kirigami-inspired patterns, and textile-based architectures, provide an effective alternative to enhance mechanical compliance without compromising electrical performance. Wang et al. [224] reported a stretchable and conformable TENG using zigzag-arranged, three-ply twisted silver-coated nylon yarn embedded in flexible silicone rubber, achieving up to 30% stretchability, high sensitivity, rapid response, and excellent mechanical stability (Fig. 6c–e). Similar engineering strategies have been applied to energy storage devices. Huang et al. [225] developed a highly stretchable and self-healable supercapacitor based on a dual-crosslinked polyacrylic acid, in which hydrogen bonding and silica nanoparticles (VSNPs–PAA) enable both large deformation and autonomous recovery (Fig. 6f), outperforming conventional polyvinyl alcohol-based electrolytes. Yu et al. [226] demonstrated a supercapacitor capable of real-time self-healing during operation, sustaining up to 800% stretchability while maintaining electrochemical performance throughout repeated charge–discharge cycles (Fig. 6g).

Beyond power generation and storage, mechanically robust optoelectronic components are also critical for next-generation flexible systems. Jang et al. [227] showed that CNT transparent electrodes significantly enhance the performance of flexible organic photodetectors by substantially reducing dark current while preserving high flexibility and optical transparency. The CNT films exhibit an average visible transmittance of 90%, achieving an effective balance between optical clarity and charge collection efficiency (Fig. 6h). These advanced, stretchable, and compressible energy systems showcase how innovative materials and design can overcome natural mechanical limits, providing durable, high-performance power solutions for flexible, long-term acoustic telemetry applications.

#### 6.1.3 Nature-inspired biodegradable flexible energy harvesting and storage systems

In addition to mechanical resilience and long-term durability, the next frontier in sustainable flexible energy systems is ensuring environmental compatibility. In marine telemetry, large-scale deployments of tags, receiver nodes, and auxiliary electronics raise concerns about retrieval and their long-term ecological effects. Nature-inspired biodegradable and bioresorbable energy-harvesting and storage devices offer a promising solution to reduce electronic waste while enabling temporary, mission-specific operations in sensitive aquatic ecosystems. The rapid growth of electronic waste and the toxic effects of many electronic materials on human health demand urgent action to support sustainable, flexible electronics development. Biodegradable and bioresorbable energy-harvesting and storage devices can significantly reduce the environmental and public health impacts of e-waste by using materials that safely dissolve, decompose, or are naturally absorbed after their functional lifespan. These eco-friendly systems aim to meet performance requirements while minimizing ecological footprints, contributing to cleaner environments and healthier ecosystems [45,46,59,64,228]. These strategies are becoming more important in acoustic telemetry, especially when devices may be lost, impossible to recover, or deployed on such a large scale that retrieval is impractical.

Biodegradable flexible energy harvesting and storage devices typically rely on three main materials: naturally derived biodegradable substances [47,233,234], soluble metals [65], and biodegradable polymers [48,235]. Together, these materials enable electronic components to degrade under external stimuli such as light, temperature, pH variation, or humidity. For example, Chen et al. [236] designed an all-wood asymmetric supercapacitor using activated wood carbon as the anode, a thin wood membrane as the separator, and MnO₂/wood carbon as the cathode (Fig. 7a). This environmentally degradable design achieved an exceptional areal capacitance of 3.6 F cm⁻², attributed to the conductive carbonized wood structure with low tortuosity. Additionally, Zhang et al. [237] developed a fully soluble and recyclable TENG capable of harvesting mechanical energy from vibrations and heartbeats. The device dissolves in water within minutes, highlighting a pathway toward eco-friendly energy harvesting systems (Fig. 7b,c).

**Fig. 7:**
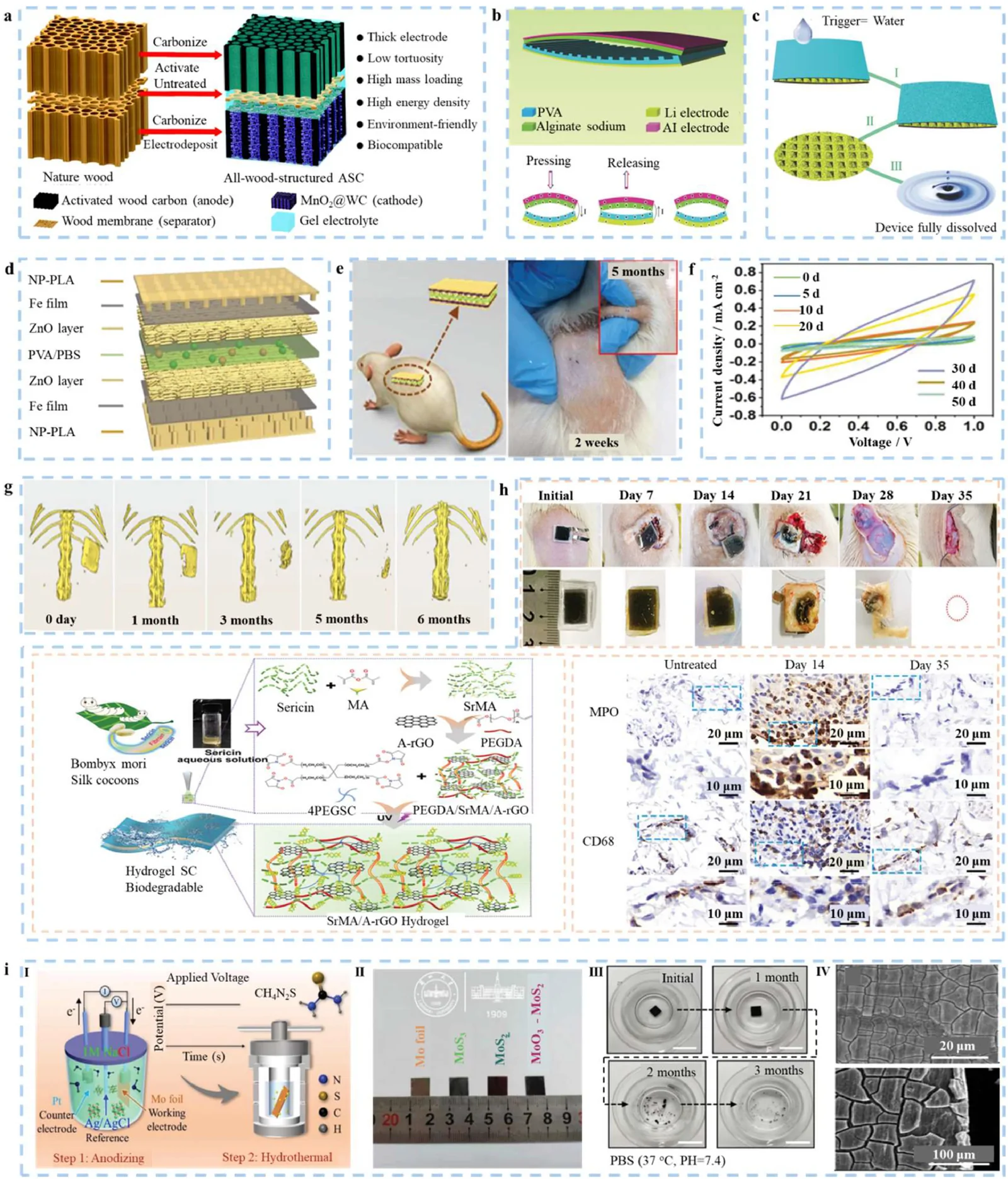
**Integrating biodegradable flexible energy harvesting with acoustic telemetry enables the development of next-generation unsinkable or self-repairing tags and receivers, expanding acoustic tracking’s temporal and spatial range.** (a) Graphical illustration of the design concept and construction process for the all-wood-structured supercapacitor [236]. Copyright 2017, Royal Society of Chemistry. (b) Diagram and dissolution mechanism of the recyclable and green TENG. (c) Dissolution process and chemical reactions during each component’s dissolution. (b and c) [237]. Copyright 2016, Wiley-VCH Verlag GmbH Co. KGaA. (d) Structure of the as-fabricated bioabsorbable supercapacitor. (e) Implantation diagram of a BC in a rat’s dorsal subcutaneous region (left) and images of the implant site before and after degradation at different times (right). (f) CV curves of an implanted BC over 50 days. (g) In vivo biodegradation of BC in SD rats over 6 months using micro-CT imaging. (d–g) [239]. Copyright 2019, Wiley. (h) (Left) Schematic of fabricating and applying the SrMA/A-rGO hydrogel supercapacitor. (Middle) Photographs of degradation assessment for implant in SD subcutaneous area. (Right) Immunohistochemical analysis images [240]. Copyright 2023, Wiley-VCH GmbH. (i-I) Schematic of MoO_3_–MoS_2_ composite electrode fabrication. (i-II) Photograph of MoO_3_−MoS_2_ composite electrode. (i-III) Optical images show MoO_3_–MoS_2_ composite electrode (11 cm^2^) dissolution in PBS (37°C, pH 7.4) at various stages. Scale bars: 2 cm. (i-IV) SEM images of MoO_3_–MoS_2_ composite electrode surface (left) and edge (right) in the dissolution process [261]. Copyright 2022, Wiley-VCH GmbH.

Bioabsorbable energy-harvesting and storage devices have also attracted significant attention in biomedical electronics, where they can eliminate secondary surgeries and reduce infection risks associated with permanent implants [42,238]. Although biomedical deployment differs from marine monitoring, these studies provide strong proof-of-concept demonstrations of safe degradation, biocompatibility, and controlled functional lifetime. Such systems often employ water-soluble transition metals such as tungsten (W), magnesium (Mg), iron (Fe), zinc (Zn), and molybdenum (Mo) as conductive electrodes or current collectors, combined with biodegradable polymers including silk, poly(1,8-octanediol-co-citrate), poly(glycerol sebacate), polylactic-co-glycolic acid, polycaprolactone, and polylactic acid (PLA). Li et al. [239] developed a fully bioabsorbable supercapacitor for lifetime implantation featuring a symmetrical layer-by-layer structure composed of PLA nanopillars, self-assembled zinc oxide nanoporous layers, and a PVA/PBS hydrogel electrolyte (Fig. 7d). In Sprague–Dawley rats, the device exhibited excellent biocompatibility and healed without inflammation after 2 weeks (Fig. 7e). It maintained in vivo performance for 50 days before gradual degradation (Fig. 7f), while micro-CT imaging confirmed complete absorption after 5 months (Fig. 7g). Similarly, Lv et al. [240] developed a supercapacitor implant using a sericin hydrogel electrode modified with aminated reduced graphene oxide and methacrylic anhydride, cross-linked using polyethylene glycol derivatives (Fig. 7h). This design provided high conductivity, mechanical flexibility, soft-tissue compatibility, and controlled biodegradation, demonstrating the feasibility of temporary high-performance energy systems.

### 6.2 Ocean wave energy harvesting for long-term telemetry deployment

While flexible self-healing and biodegradable energy systems enhance the resilience and sustainability of telemetry hardware, long-term deployment ultimately relies on energy autonomy. Ocean wave energy is among the most abundant and consistently available renewable energy sources in marine environments, making it a promising means of extending the lifetimes of offshore telemetry nodes, receiver buoys, and future autonomous acoustic monitoring arrays. Ocean waves represent a vast renewable energy resource, as oceans cover about 71% of Earth’s surface [60], making wave energy a promising option for clean, sustainable power generation. However, harnessing wave energy remains challenging, particularly for compact and low-maintenance systems. Traditional electromagnetic generators (EMGs), widely used in nearshore and offshore power plant applications, are often bulky, costly, and mechanically complex [44], limiting their suitability for small-scale marine sensors and autonomous observing systems. These limitations are directly relevant to acoustic telemetry, where long-term receiver deployments and surface relay platforms are often constrained by battery capacity, maintenance requirements, and the high operational cost of retrieval and redeployment.

In recent years, lightweight TENGs have emerged as a promising alternative, offering high power density and strong efficiency at the low, irregular frequencies of ocean waves. TENGs convert ambient mechanical energy into electricity [37,[241], [242], [243]], making them attractive for autonomous sensor networks [90,[244], [245], [246], [247]] and marine energy harvesting applications [38,39,[248], [249], [250], [251], [252], [253]], due to their compact size, scalability, and adaptability. Importantly, wave-driven energy harvesting aligns well with the needs of long-term acoustic telemetry infrastructure (e.g., fixed receivers, gateway buoys, or acoustic relay nodes), where continuous low-power energy input can extend operational lifetimes, reduce servicing frequency, and support persistent monitoring.

Hybrid generators combining TENGs and EMGs further improve energy conversion efficiency by optimizing wave-body interactions and device architecture, thereby increasing output and improving adaptability to ocean conditions [50,254]. Omnidirectional designs, such as multi-track spherical TENGs (Fig. 8a) and gyroscope-shaped TENGs (Fig. 8b,c), enhance multidirectional energy harvesting while improving stability and durability in complex marine environments [255,256]. Fluid oscillation-driven systems, such as oscillating water column TENGs, enhance low-frequency wave energy capture for powering small offshore sensors [257]. While traditional piezoelectric methods face efficiency limitations in low-frequency marine environments, recent strategies, like hydro-cavitation, can increase local pressure and enhance electrical output [258]. Long-term field tests demonstrate durability and practicality, with some devices extending buoy lifetime and enabling real-time wave sensing [50,259]. Overall, these developments support the gradual transition of ocean wave energy harvesting from laboratory prototypes toward scalable marine sensing platforms and distributed ocean networks [36,39,260]. Cheng et al. [198] introduced a soft-contact spherical TENG (SS-TENG) that improved output by optimizing materials and structural design (see Fig. 8d). It uses a flexible liquid/silicone core inside an acrylic sphere, replacing the hard PTFE core. The soft core enhances contact during motion, increasing charge transfer, while tunable core softness allows adaptation to different wave conditions.

**Fig. 8:**
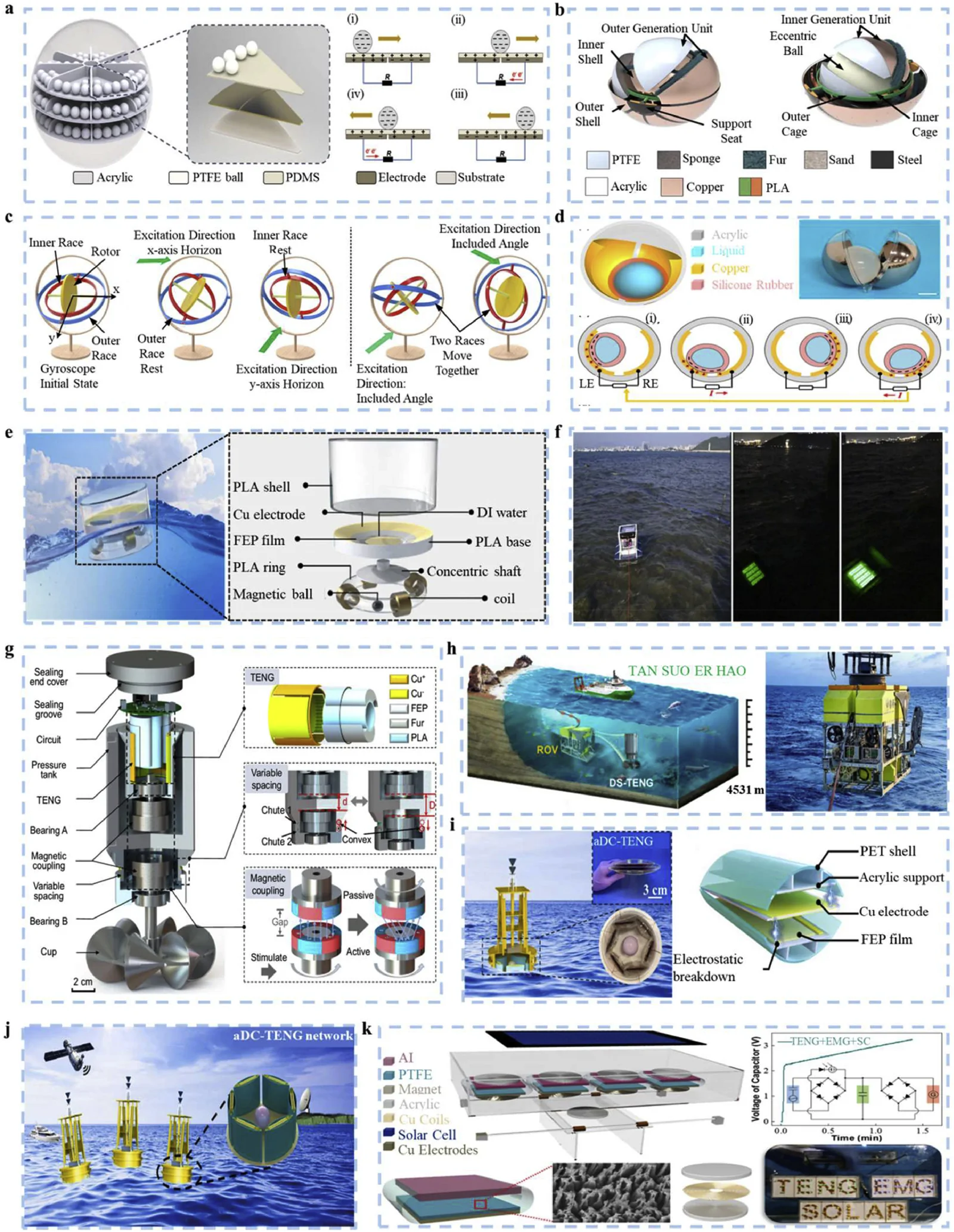
**Ocean wave energy harvesting technologies for long-term tracking devices.** (a) Schematic of the omnidirectional multi-track spherical TENG (OMS-TENG) based on an eight-track fan-type TENG (FTENG) and its working mechanism [255]. Copyright 2024, Wiley-VCH GmbH. (b) Structural design of gyroscope-structured TENG for harvesting multidirectional ocean energy (GS-TENG). (c) Operating mechanism of the GS-TENG under excitation from different directions. (b and c) [256]. Copyright 2022, American Chemical Society. (d) Schematic and working mechanism of the soft-contact spherical TENG (SS-TENG) [198]. Copyright 2019, Elsevier. (e) Schematic of the fabricated guided-liquid-based, iTEHG. (f) Photographs show 320 green power LEDs lit instantly and continuously by a single iTEHG in Sanya Bay, China. (e and f) [31]. Copyright 2023, Elsevier. (g) An exploded view of the TENG-based fully integrated, self-powered, ultra-sensitive ocean current sensor (left). Three-dimensional schematics show magnetic coupling (right bottom), variable spacing (right center), and TENG (upper right). (h) Schematic of deep-sea experiments and DS-TENG application (left). DS-TENG integrated with ROV (right). (g and h) [40]. Copyright 2024, Springer Nature. (i) Schematic of aDC-TENG array with ocean buoy. Insets show a photo of aDC-TENG above an array (left) and a 3D schematic of a single unit (right). (j) Design schematic of the aDC-TENG network for large-scale ocean wave energy harvesting. (i and j) [32]. Copyright 2024, Elsevier. (k) Structure design of the hybrid TENG for harvesting blue energy includes a four-cycle contact-separate TENG (CS-TENG), a freestanding sliding EMG (FS-EMG), and a commercial waterproof silicon solar cell (WS-SC). [262]. Copyright 2017, Elsevier.

Recent advances in blue energy harvesting have led Xu et al. [31] to develop a guided-liquid isotropic triboelectric–electromagnetic hybrid nanogenerator (iTEHG), designed to efficiently capture wave energy from all directions (Fig. 8e). It uses EMG effects to feature a concentric circular electrode pair in a solid-liquid-interfaced TENG on a tilted satellite-dish-inspired platform. This design addresses wear and contact degradation common in traditional solid–solid TENGs, thereby improving device longevity, an important requirement for long-term marine deployments. Additionally, gravity-guided liquid motion enhances controllability and stabilizes output under irregular excitation. The system achieved uniform power and current densities of 7.25 μW cm^–3^ and 4.85 μA cm^–3^, with <5% variation across different directions. It can charge a 0.1 F supercapacitor to 3.1 V in 30 min, powering temperature and humidity sensors for over 26 min. Tested in lab and during sea trials in Sanya Bay, China, the device successfully lit 320 LEDs at high brightness under multidirectional wave conditions, even with low ocean activity (Fig. 8f). These demonstrations highlight the potential of hybrid TENG-EMG platforms as robust power supplies for long-term ocean observation systems and telemetry-supporting infrastructures.

Recent advances in marine sensing have enabled transformative deep-sea technologies. Pan et al. [40] introduced a fully integrated, self-powered, high-pressure-resistant, ultra-sensitive ocean current monitoring system (DS-TENG), shown in Fig. 8g. It measures flow velocities at incredible depths up to 4531 m, inspired by biological fur and flexible foils that harvest energy from currents and sense flow parameters. The DS-TENG design reduces structural friction while improving sensitivity and durability. A magnetic coupling mechanism enables non-contact energy transfer, generating flow-induced electrical signals without an external power supply. The device incorporates an adjustable mechanism that supports velocity measurements from 0.02 to 6.69 m/s, representing a 67% improvement over conventional vertical-axis current sensors. Deployed at 4531 m in the South China Sea (Fig. 8h), it operated continuously for 6 h, monitoring currents between 0.16–0.76 m/s. These results demonstrate the feasibility of self-powered sensing systems in extreme environments, which could be extended to support future deep-sea telemetry receiver arrays and autonomous acoustic monitoring networks where battery replacement is impractical.

The persistent challenge of harvesting energy from low-frequency, irregular ocean waves continues to drive the development of innovative TENG architectures. Feng et al. [32] developed a pulsed arch-shaped double-layered direct-current TENG (aDC-TENG) designed specifically for ultra-low-frequency ocean wave energy harvesting (Fig. 8i). The aDC-TENG incorporates an optimized arch structure to reduce mechanical resistance and improve durability, along with a distributed-electrode design that produces a unidirectional current output. The device achieved a current of 23.5 μA at an ultra-low triggering frequency of 0.1 Hz and demonstrated an instantaneous power density of 0.25 W m^–2^, sufficient to light 230 LEDs in laboratory testing. In marine field trials (Fig. 8j), when integrated at the base of an ocean buoy, it successfully harvested omnidirectional wave energy from real ocean conditions. The harvested energy proved sufficient to power 50 LEDs and activate the boy’s warning system. These results highlight the feasibility of integrating wave-driven energy harvesters into surface platforms that could serve as acoustic telemetry gateways, powering receivers, communication modules, and onboard processing units for real-time data relay.

Overall, ocean wave energy harvesting, particularly through robust TENG and hybrid TENG–EMG architectures, offers a promising pathway toward maintenance-free, long-term marine telemetry deployment. As these systems mature, their integration with acoustic telemetry infrastructure may enable persistent receiver arrays, autonomous surface gateways, and self-powered relay platforms, reducing dependence on battery replacements. These advances support the broader vision of scalable, long-duration marine monitoring networks capable of operating across large spatial and temporal scales.

### 6.3 Ecological impacts of emerging energy and material technologies

Emerging technologies, such as flexible, self-healing, biodegradable materials and energy-harvesting systems, provide benefits for long-term, autonomous acoustic telemetry [32,50,198]. However, their ecological impacts need thorough assessment. Biodegradable materials could reduce electronic waste, but their degradation and ecological effects require more research [263,264]. Future studies should incorporate environmental impact assessments in the design of next-generation telemetry systems. Consequently, we examine ecological implications across three levels: animal welfare, habitat effects, and the material life cycle.

From an animal welfare perspective, recent advances in flexible, miniaturized tags aim to reduce mechanical stress, physical burden, and behavioral disturbance while enhancing hydrodynamic performance. Matley et al. [265] synthesized 149 electronic tagging studies to examine tag burden and long-term impacts, finding limited evidence of long-term negative effects but a lack of assessments over months, leaving impacts on foraging, reproduction, and physiology poorly understood and needing future study. Yang et al. [90] developed a miniaturized tag called the “Lab-on-a-fish” to monitor ECG, EMG, activity, and environmental conditions for up to 8 months in relatively small fish. However, it requires surgical implantation and long deployments, necessitating careful validation of healing, behavior, and survival to ensure that tagging does not affect natural behavior or bias ecological interpretations. Conversely, Nassar et al. [30] developed a non-invasive, conformal, ultralight skin tag called “Marine Skin” that attaches externally to crustaceans to record depth, temperature, and salinity. These tags match the body shape, minimize drag and handling impacts, and provide a more humane and eco-friendly monitoring method. Additionally, acoustic telemetry is used in aquaculture as a welfare tool, with accelerometer tags detecting behavioral responses to hypoxia, ammonia, temperature, water quality, and cage conditions, offering real-time welfare data [[266], [267], [268], [269]].

At the habitat and community level, acoustic telemetry interacts with marine soundscapes and infrastructure. Although telemetry itself is generally low-impact, related factors such as vessel activity, moored platforms, and acoustic emissions can affect animal movement and habitat use. Acoustic tracking is used to assess how shipping and human activities influence movement, habitat use, and communication, impacting community structure and dependent predators [4,197,270,271]. For instance, shipping noise displaced Arctic cod home ranges, altered their behavior during key feeding times, and posed risks to seabirds, marine mammals, and Indigenous users [197]. More broadly, anthropogenic noise has been shown to influence species abundance, foraging behavior, and community composition. These findings highlight the need to ensure that telemetry deployments, particularly large-scale or long-term deployments, do not cause acoustic disturbance or alter ecological interactions [271].

From a materials and life-cycle perspective, the environmental impact of telemetry systems is growing, especially as large-scale ocean observation networks expand. Life-cycle assessments of electronic and IoT devices show that manufacturing, particularly batteries and electronic components, accounts for most emissions and ecotoxicity [272]. To address this, new research on flexible, biodegradable electronics proposes using bio-derived polymers, recyclable substrates, and carbon-based conductors to reduce environmental impact. For instance, replacing traditional materials with bio-based or recyclable alternatives can lower global warming potential by 30%–40% or more [263,264,273]. In acoustic telemetry, these insights enable the development of biodegradable, self-healing energy sources, such as triboelectric nanogenerators, and bioresorbable storage solutions, reducing environmental accumulation and the need for retrieval. However, more studies are necessary to understand degradation processes, long-term stability, and ecological safety in real marine environments.

Overall, emerging technologies enable more sustainable and autonomous acoustic telemetry systems, but their ecological effects require careful evaluation through interdisciplinary methods that integrate engineering, animal physiology, and ecosystem science.

## 7 Conclusion

Acoustic telemetry has transformed the study of aquatic animals and ecosystems by enabling detailed, long-term observations that are essential for marine science, conservation, and fisheries management. However, the lifespan and scalability of telemetry infrastructure, particularly receiver arrays, relay platforms, and autonomous monitoring systems, remain constrained by battery limitations, harsh environmental conditions, and the logistical cost of maintenance in remote ocean regions. To address these challenges, emerging energy technologies are opening new pathways to sustainable, autonomous telemetry deployments. Flexible and bio-inspired energy storage systems, including self-healing, stretchable, and biodegradable devices, offer improved durability, safety, and environmental compatibility. In parallel, ocean energy-harvesting approaches, such as triboelectric, piezoelectric, and hybrid triboelectric-electromagnetic generators, have demonstrated strong potential to power long-term marine sensor nodes under low-frequency, multidirectional wave conditions, thereby enabling self-powered, maintenance-reduced operation.

This review summarizes the principles and system components of acoustic telemetry, highlights its expanding applications in pollution monitoring, climate change assessment, marine protected area planning, and combating illegal, unreported, and unregulated fishing, and discusses key technical and biological limitations. Unlike previous reviews, this work emphasizes how integrating acoustic telemetry with sustainable energy technologies can strengthen long-term monitoring capacity, reduce operational constraints, and improve resilience for next-generation ocean observation networks. Continued progress in energy efficiency, materials engineering, system integration, and deployment design will be essential to maximize the role of acoustic telemetry in marine research, biodiversity conservation, and sustainable ocean resource management.

## CRediT authorship contribution statement

**Abdelrahman Samy Ismail:** Conceptualization, Investigation, Visualization, Writing – original draft, Writing – review & editing. **Zhenting Ye:** Investigation, Visualization, Writing – review & editing. **Amar N. Alsheavi:** Visualization. **Yaxiaer Yalikun:** Investigation, Writing – review & editing. **Yang Yang:** Conceptualization, Investigation, Supervision, Funding acquisition, Writing – review & editing. **Chenjing Shang:** Conceptualization, Investigation, Supervision, Funding acquisition, Writing – review & editing.

## Declaration of competing interest

The authors declare that they have no conflicts of interest in this work.
